# PARP inhibition impedes the maturation of nascent DNA strands during DNA replication

**DOI:** 10.1038/s41594-022-00747-1

**Published:** 2022-03-24

**Authors:** Alina Vaitsiankova, Kamila Burdova, Margarita Sobol, Amit Gautam, Oldrich Benada, Hana Hanzlikova, Keith W. Caldecott

**Affiliations:** 1 Genome Damage and Stability Centre, School of Life Sciences, University of Sussex, Brighton, UK; 2 Laboratory of Genome Dynamics, Institute of Molecular Genetics of the Czech Academy of Sciences, Prague 4, Czech Republic; 3 Laboratory of Molecular Structure Characterization, Institute of Microbiology of the Czech Academy of Sciences, Prague 4, Czech Republic

**Keywords:** Single-strand DNA breaks, DNA synthesis

## Abstract

Poly(ADP-ribose) polymerase 1 (PARP1) is implicated in the detection and processing of unligated Okazaki fragments and other DNA replication intermediates, highlighting such structures as potential sources of genome breakage induced by PARP inhibition. Here, we show that PARP1 activity is greatly elevated in chicken and human S phase cells in which FEN1 nuclease is genetically deleted and is highest behind DNA replication forks. PARP inhibitor reduces the integrity of nascent DNA strands in both wild-type chicken and human cells during DNA replication, and does so in *FEN1*^*−/−*^ cells to an even greater extent that can be detected as postreplicative single-strand nicks or gaps. Collectively, these data show that PARP inhibitors impede the maturation of nascent DNA strands during DNA replication, and implicate unligated Okazaki fragments and other nascent strand discontinuities in the cytotoxicity of these compounds.

## Abstract (web summary)

Biochemical and cell-based assays reveal that PARP inhibitors impede the maturation of nascent DNA strands during DNA replication, and implicate unligated Okazaki fragments and other nascent strand discontinuities in the cytotoxicity of these anti-cancer compounds.

## Main

Poly(ADP-ribose) polymerases (PARPs) are a superfamily of enzymes that use NAD^+^ to modify themselves and other proteins with mono- or poly(ADP-ribose)^1,2^. The archetypal PARP enzyme is PARP1 that, along with PARP2 and PARP3, is activated by DNA breaks and regulates the cellular DNA damage response^3–5^. Poly(ADP-ribose) is a highly dynamic and transient signal that is rapidly degraded by poly(ADP-ribose) glycohydrolase (PARG)^6–8^. DNA damage-stimulated PARPs bind to and are activated by a variety of DNA substrates, of which DNA single-strand breaks (SSBs) and double-strand breaks are the best characterized. At SSBs, PARP1 and PARP2 fulfill a variety of roles, depending on the nature and source of the break, including the regulation of chromatin compaction and the recruitment of DNA repair proteins^5,9^.

In addition to DNA breaks arising stochastically across the genome, PARP1 and PARP2 are also involved in the detection and processing of various DNA replication intermediates^10^. Indeed, S phase is the primary source of poly(ADP ribosylation) in unperturbed proliferating cells^11^. For example, PARP1 may detect and signal the presence of paused, reversed and/or collapsed DNA replication forks^12,13^. A likely role for PARP1 and/or PARP2 at collapsed forks is to suppress binding by Ku and 53BP1, which otherwise can trigger ‘toxic’ nonhomologous end-joining (NHEJ)^14–16^. In addition, PARP activity may promote homologous recombination (HR)-mediated resetting and/or repair of reversed or collapsed forks, by regulating the recruitment and/or activity of MRE11 nuclease^12,17^. PARP1 can also regulate the longevity of reversed forks by inhibiting RECQ1, a helicase that can reset reversed forks independently of RAD51-mediated HR^18,19^.

Recently, we have implicated PARP1 in the detection of unligated Okazaki fragments^11^. The synthesis of Okazaki fragments is initiated by DNA polymerase α-primase complex (POLα), which generates short RNA primers that are extended by POLα for 10–20 deoxyribonucleotides followed by DNA polymerase δ (POLδ) for approximately a further 200 deoxyribonucleotides, until the 5′ terminus of the downstream Okazaki fragment is encountered^20–24^. The junctions between adjacent fragments are then processed and ligated by flap endonuclease-1 (FEN1) and DNA ligase I (LIG1), respectively, although other nucleases can be involved^20–25^. While this canonical pathway for the maturation of Okazaki fragments is very efficient, it has been estimated from biochemical experiments that roughly 15–30% of human POLδ molecules disengage before reaching a downstream Okazaki fragment, even in the presence of the proliferating cell nuclear antigen (PCNA) processivity factor^26^. Given that each human S phase entails the formation of 30–50 million Okazaki fragments, failure of the canonical pathway to ligate even just 0.1% of Okazaki fragment intermediates would result in 30,000–50,000 SSBs and/or single-strand gaps each S phase. PARP1-dependent signaling and repair may thus help ensuring the integrity of nascent DNA strands during DNA replication^10^. Consistent with this idea, SSB repair proteins recruited at DNA breaks by PARP1 such as X-ray repair cross-complementing protein 1 (XRCC1) and DNA ligase III (LIG3) have been associated with Okazaki fragment maturation^11,27–29^.

Inhibitors of PARP are clinically approved drugs for the treatment of cancer cells in which HR-mediated repair is defective, based on of their extreme toxicity in such cells^30–32^. A critical mechanistic aspect of such inhibitors is their ability to ‘trap’ PARP enzymes on their DNA substrates, which in the absence of efficient HR-mediated repair leads to cell death^32,33^. However, the endogenous DNA substrates on which PARP becomes trapped and the impact of this trapping on DNA replication are unclear, with intermediates of base and ribonucleotide excision repair (RER), single-strand gaps and stalled/broken DNA replication forks all possible contributors^34–38^. Here we have further addressed this question, and show that PARP inhibitors impede the maturation of nascent DNA strands during DNA replication, and that intermediates of Okazaki fragment processing are likely to be a major source of cytotoxic PARP1 trapping.

## Results

### PARP activity and PARP inhibitor sensitivity in *FEN1*^*−/−*^ DT40

**Fig. 1:**
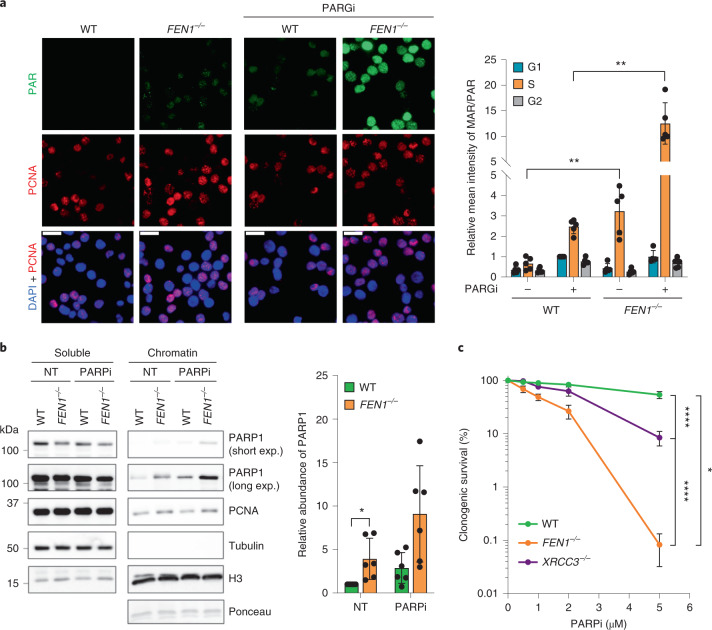
Increased PARP1 activity and sensitivity to PARP inhibitor in *FEN1*^***−*/*−***^ DT40 cells. **a**, Representative images (left) and scanR quantification (right) of ADP-ribose detected by the PAR-specific detection reagent (MABE1031) or ADP-ribose (MAR/PAR) mAb E6F6A (CST 83732), respectively, in WT and *FEN1*^*−/−*^ DT40 cells. Where indicated, cells were incubated with 10 µM PARG inhibitor (PARGi) for 30 min to prevent poly(ADP-ribose) degradation. Data are the mean (±s.d.) ADP-ribose fluorescence normalized to that in PARGi-treated WT cells in G1, from five independent experiments (individual data points also plotted). Cell cycle stage was distinguished by EdU pulse labeling (10 µM, 30 min) and DNA content (DAPI intensity). Scale bars, 20 µm. Statistical significance was assessed by one-way analysis of variance (ANOVA) with post hoc Sidak’s multiple comparisons test (**P < 0.01). Galleries of representative cells from scanR microscopy are shown in Extended Data Fig. 1b). **b**, Western blots (left) and quantification (right) of PARP1, PCNA, tubulin and histone H3 (H3) in soluble and chromatin-containing fractions of WT and *FEN1*^*−/−*^ DT40 cells following detergent extraction. Where indicated, cells were incubated or not (NT) for 30 min with 10 µM PARP inhibitor (PARPi, Olaparib) before fractionation. For quantification (right), PARP levels in chromatin were normalized to ponceau S-stained histone levels and expressed relative to the PARP1 level in untreated WT chromatin. Data are the mean (±s.d.) of six independent experiments with individual data points plotted. Statistically significant differences (*P < 0.05) between WT and *FEN1*^*−/−*^ are shown, as determined by Kruskal–Wallis one-way ANOVA, with post hoc comparisons. **c**, Clonogenic survival of WT, *FEN1*^*−/−*^ and *XRCC3*^*−/−*^ DT40 cells incubated continuously in media containing the indicated concentrations of PARPi (Olaparib). Data are the mean (±s.d.) of four independent experiments. Statistical significance was assessed by two-way ANOVA with post hoc Tukey’s multiple comparisons test (*P < 0.05; ****P < 0.0001). Source data

**Extended Data Fig. 1:**
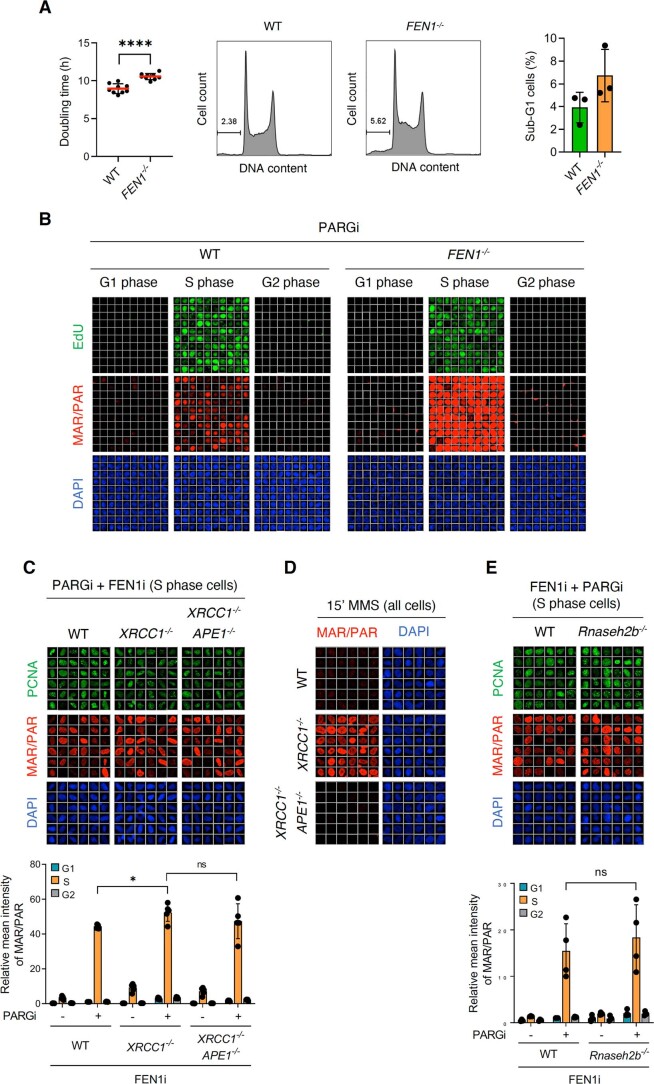
Increased doubling time and S phase poly(ADP-ribose) in *FEN1*^*-/-*^ DT40 cells. **(a)** *Left*, individual data points and mean (±SD) doubling time for wild type (WT) and *FEN1*^*-/-*^ DT40 cells (n = 9). Statistical significance assessed by two-tailed paired t test (****P < 0.0001). *Middle*, representative cell cycle distributions. *Right*, Fraction of cells with sub-G1 content. Data are the mean (±SD, n = 3) with individual data points also plotted. **(b)** Representative scanR galleries of data in Fig. 1a. ADP-ribose (MAR/PAR) was detected using rabbit mAb E6F6A (CST 83732) in WT and *FEN1*^*-/-*^ DT40 cells (only PARGi-treated cells are shown). **(c)** Representative scanR galleries (*top*) and quantification (*bottom*) of ADP-ribose detected as above in WT, *XRCC1*^*-/-*^, and *XRCC1*^*-/-*^*/APE1*^*-/-*^ RPE-1 cells treated for 90 min + /−20 µM FEN1 inhibitor (FEN1i). Where indicated 10 µM PARG inhibitor (PARGi) was present during the last 30 min. Cell cycle phase was distinguished by PCNA staining and DNA (DAPI) content. Mean ADP-ribose fluorescence was normalised to WT RPE-1 cells in G1 treated with both FEN1i and PARGi. Data are the mean (±SD) normalised ADP-ribose intensity (n = 5) with individual data points plotted. Statistical significance assessed by 1-way ANOVA with post hoc Sidak’s multiple comparisons test (ns, not significant, *p < 0.05). APE1 deletion was combined with XRCC1 deletion to increase SSB half-life and assay sensitivity. **(d)** Representative scanR galleries of ADP-ribose detected as above following 15 min with 0.01% MMS in WT, *XRCC1*^*-/-*^, and *XRCC1*^*-/-*^*/APE1*^*-/-*^ RPE-1 cells (to confirm APE1 loss). **(e)** Representative scanR galleries (*top*) and quantification (*bottom*) of ADP-ribose detected as above in WT and *Rnaseh2b*^*-/-*^ MEFs treated for 90 min with 20 µM FEN1 inhibitor (FEN1i). Where indicated, 10 µM PARG inhibitor (PARGi) was present during the last 30 min. Cell cycle analysis/presentation as in panel C. Data are the mean (±SD) ADP-ribose intensity (n = 3–4) with individual data points also plotted. Significance assessed by two-tailed paired t test. Source data

To examine the possibility that PARP inhibitors might ‘trap’ PARP1 on unligated Okazaki fragments, we used chicken DT40 cells in which FEN1 was deleted by gene targeting^29^. *FEN1*^*−*^^/^^*−*^ DT40 cells are viable and proliferate, albeit with a roughly 20% increased doubling time due at least in part to increased cell death (ref. ^29^ and Extended Data Fig. 1a), suggesting that alternative pathways for Okazaki fragment processing must operate in these cells. Indeed, other nucleases can function during Okazaki fragment processing, as can PARP1-dependent DNA SSB repair (SSBR)^11,27,28,39–42^. Consistent with an involvement of the latter pathway, short incubation with PARG inhibitor to preserve nascent poly(ADP-ribose) uncovered elevated levels of ADP ribosylation in *FEN1*^*−/−*^ DT40 cells, specifically in S phase (Fig. 1a and Extended Data Fig. 1b). In fact, elevated S phase ADP ribosylation was also detected in *FEN1*^*−*^^*/*^^*−*^ DT40 cells in the absence of PARG inhibition (Fig. 1a). These data are consistent with our previous finding that S phase ADP ribosylation is greatly increased in human cells by incubation with FEN1 inhibitor^11^.

In addition to Okazaki fragment processing, FEN1 is involved in several DNA excision repair pathways during S phase that could contribute to the elevated ADP ribosylation in FEN1-defective cells such as DNA base excision repair (BER) and RER^43,44^. The latter pathway is of particular interest, because of the prevalence of ribonucleotides during the S phase and because of the impact of ribonucleotide excision on PARP1 activation^35^. However, genetic deletion of neither APE1 nor RnaseH2 to suppress these excision repair pathways affected the level of S phase ADP ribosylation induced by FEN1 inhibitor (Extended Data Fig. 1c–e), which is consistent with our previous report^11^ that neither BER nor RER contribute greatly to the overall level of endogenous S phase PARP activity.

The amount of PARP1 present in the detergent-insoluble fraction of *FEN1*^*−*^^*/*^^*−*^ DT40 cells was also elevated when compared to wild-type (WT) cells, and was increased further by PARP inhibitor, consistent with the ‘trapping’ of PARP1 on unligated Okazaki fragments (Fig. 1b). Notably, *FEN1*^*−/−*^ DT40 cells were more sensitive to PARP inhibitor than were *XRCC3*^*−/−*^ DT40 cells that lack efficient HR-mediated repair, which is the archetypal determinant of cellular sensitivity to PARP inhibitors, suggesting that PARP1 trapping on Okazaki fragments is a highly toxic event (Fig. 1c).

### PARP inhibition and nascent strand integrity

**Fig. 2:**
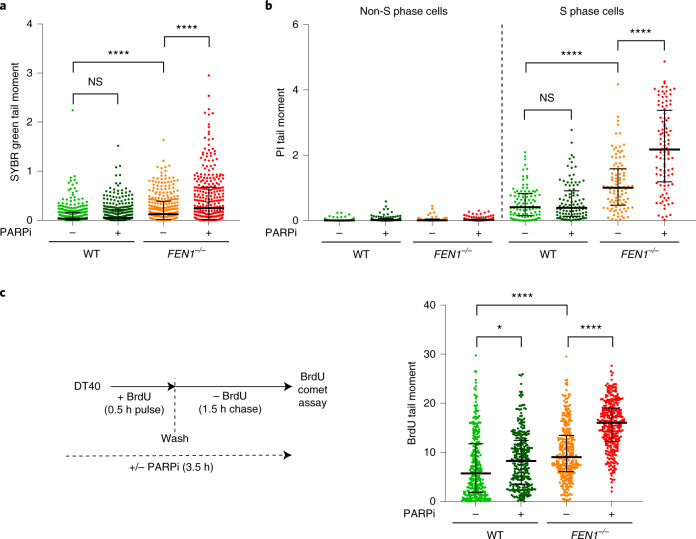
PARP inhibitor impedes the maturation of large/late nascent DNA strands in WT and *FEN1*^*−/−*^ chicken DT40 cells. **a**, DNA breaks in genomic DNA quantified by alkaline comet assays in WT and *FEN1*^*−/−*^ DT40 cells following incubation (2 h) or not with PARPi (10 µM KU0058948). Comet tail moments (an arbitrary unit of DNA breaks) were scored following staining of genomic DNA with SYBR Green. For each sample, scatter plots are the tail moments of 300 individual cells combined from *n* = 3 experiments (100 cells per sample per experiment) and the bars represent the median and interquartile range (see Extended Data Fig. 2a for individual experimental data sets). Statistical significance was determined from mean tail moments (*n* = 3) by two-way ANOVA with Tukey’s post hoc tests (NS, not significant; *P < 0.05; ****P < 0.0001). **b**, DNA breaks in total genomic DNA quantified in S phase and non-S phase cells following incubation (2 h) or not with PARPi (10 µM Olaparib). S phase cells were identified by pulse labeling (final 45 min) with BrdU. Alkaline comet tail moments were scored following staining of genomic DNA with propidium iodide (PI). For each sample, scatter plots are the tail moments of 100 individual cells combined from two independent experiments (50 cells per sample per experiment) and the bars represent the median and interquartile range (see Extended Data Fig. 2b for individual data sets). Statistics as in **a**. **c**, DNA breaks quantified in nascent DNA strands of the indicated cells following incubation or not with PARPi (10 µM Olaparib) (see schematic, left). Alkaline comet tail moments were scored in nascent single strands by staining with anti-BrdU antibodies following a 0.5 h BrdU pulse label and subsequent 1.5 h chase (‘BrdU comet tail moment’). For each sample, scatter plots show BrdU tail moments from 300 cells combined from three independent experiments (100 cells per sample per experiment) and bars are the median and interquartile range (see Extended Data Fig. 2d for individual data sets). Statistics as in **a**. Source data

**Extended Data Fig. 2:**
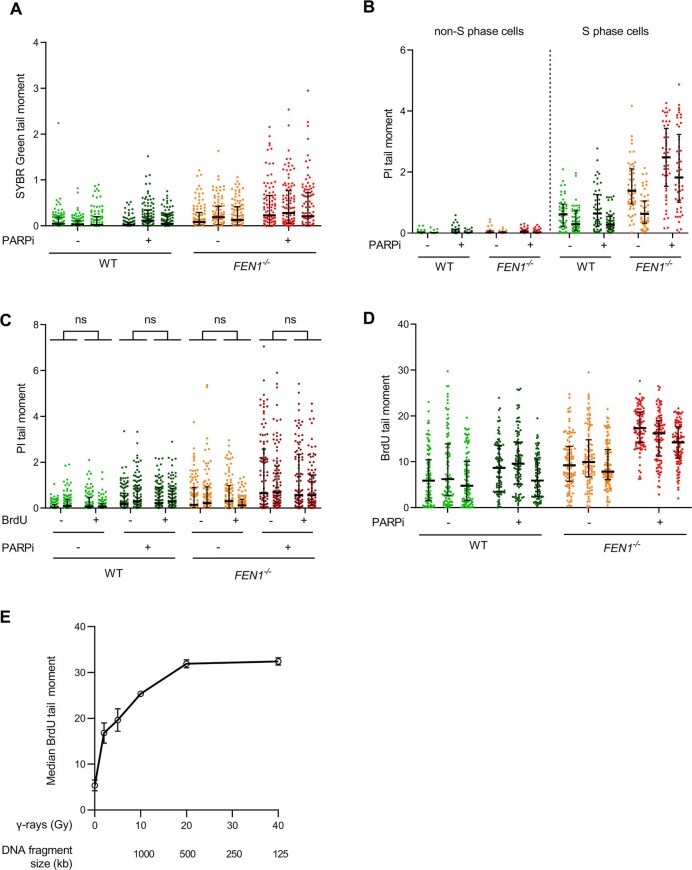
PARP inhibitor reduces the integrity of large/late nascent DNA strands in wild type and *FEN1*^*-/-*^ chicken DT40 cells. **(a, b)** Scatter plots of the individual experiments comprising Fig. 2a (n = 3) & Fig. 2b (n = 2), respectively, with each repeat plotted side-by-side. Bars are median and interquartile range. **(c)** DNA strand breaks quantified by alkaline comet assays in WT and *FEN1*^*-/-*^ DT40 cells pulse-labelled or not with BrdU for the last 45 min of a 2 h-incubation + /−10 µM PARPi (Olaparib). Genomic DNA was visualized for scoring by staining with propidium iodide (PI). Data are the individual comet tail moments from two independent experiments (100 cells/sample/experiment) plotted side by side and bars are the median and interquartile range. Statistical significance was determined from the mean tail moments of the n = 2 experiments by 2-way ANOVA with Tukey’s post hoc tests (ns, not significant). **(d)** Scatter plots of the individual experimental data sets that comprise Fig. 2c with each experimental repeat (n = 3) plotted side by side. Bars represent the median and interquartile range. **(e)** γ-ray calibration curve for BrdU alkaline comet assays. Wild type DT40 cells were incubated with media containing BrdU (100 µM) for 20 h to fully label genomic DNA and then treated with the indicated dose of γ-rays. Alkaline comet tail moments of anti-BrdU stained DNA were quantified under the same experimental conditions as those applied in BrdU pulse-labelled cells in Fig. 2c. The average (±1 SD) of the median comet tail moments from n = 2 experiments (50 cells/sample/experiment) are plotted. Average fragment sizes of single-stranded DNA following the indicated γ-ray dose is indicated, calculated on the assumption 1 Gy induces ~1100 total DNA breaks (~1000 SSBs & ~50 DSBs) per diploid human genome (~12 × 10^9^ nucleotides) and thus ~1 break every 1 × 10^7^ nucleotides. Note that BrdU alkaline tail moments are insensitive to median fragment sizes <500 kb, likely because these are lost during alkaline lysis and/or electrophoresis. Source data

To examine directly whether PARP inhibitors might block the maturation of Okazaki fragments we measured the integrity of genomic DNA in WT and *FEN1*^*−/−*^ DT40 cells using alkaline comet assays. The level of endogenous DNA breaks was roughly twofold higher in *FEN1*^*−/−*^ cells than in WT cells, as measured by their comet tail moments (an arbitrary measure of DNA strand breaks), and this difference was increased roughly by a further twofold by incubation with PARP inhibitor (Fig. 2a and Extended Data Fig. 2a). To confirm that the elevated DNA breaks in *FEN1*^*−/−*^ cells were associated with DNA replication we measured the integrity of genomic DNA specifically in S phase. Indeed, most of the DNA breaks detected by alkaline comet assays were in S phase in both WT and *FEN1*^*−/−*^ cells (Fig. 2b and Extended Data Fig. 2b). Once again, the level of endogenous DNA breaks was roughly twofold higher in *FEN1*^*−/−*^ cells than in WT cells, and was elevated by roughly a further twofold by PARP inhibitor (Fig. 2b and Extended Data Fig. 2b). The DNA breaks detected in S phase cells were not an artifact of pulse labeling S phase cells with bromodeoxyuridine (BrdU), because omission of this nucleoside from experiments had no impact on the results of our alkaline comet assays (Extended Data Fig. 2c).

To explore whether the impact of PARP inhibitor and/or FEN1 deletion in alkaline comet assays involved reduced integrity of nascent DNA strands, we pulse-labeled cells with BrdU and measured the size of the labeled DNA in alkaline comet assays using anti-BrdU antibodies (Fig. 2c). Since DNA strands are separated during alkaline comet assays, the quantification of tail moments specifically in BrdU-labeled DNA measures the size of only the nascent strands. Notably, nascent strand integrity was significantly reduced in *FEN1*^*−/−*^ DT40 cells when compared to WT cells following a 30-min BrdU pulse label and subsequent 90 min chase, consistent with a reduced rate of DNA maturation in the mutant cells during DNA replication (Fig. 2c and Extended Data Fig. 2d). Moreover, PARP inhibitor exacerbated the impact of FEN1 deletion in these experiments, and even reduced the integrity of nascent strands in WT DT40 cells (Fig. 2c and Extended Data Fig. 2d).

**Fig. 3:**
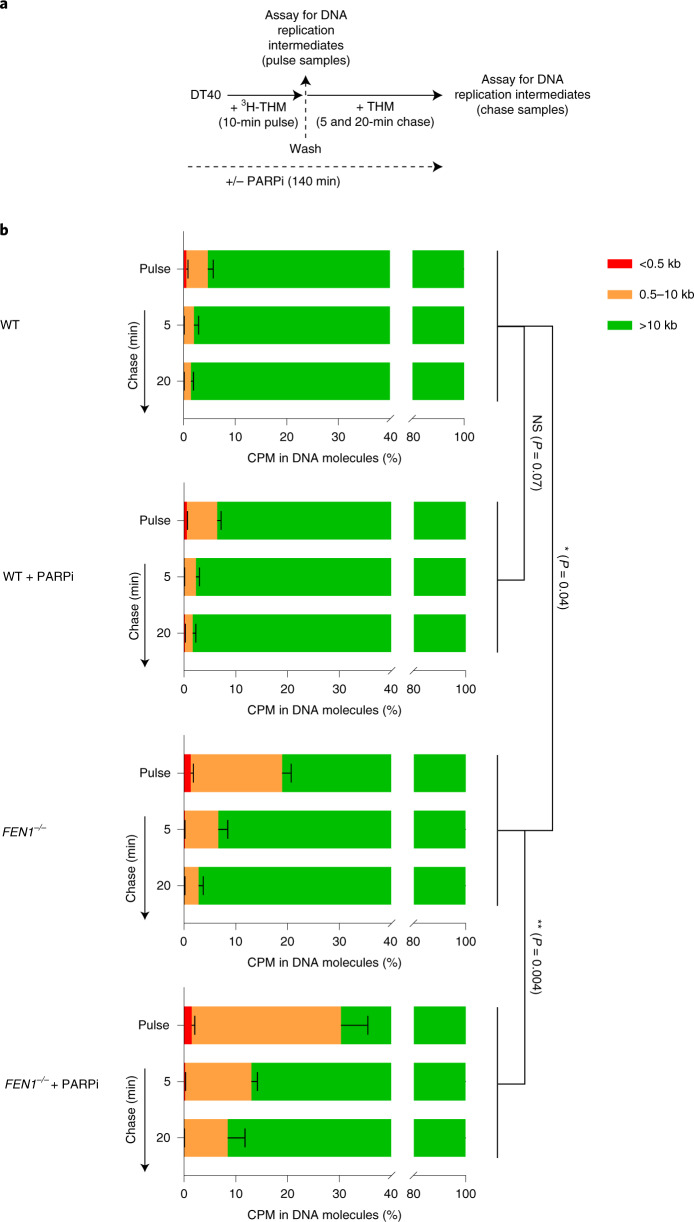
PARP inhibitor impedes the maturation of early/small (<10 kb) nascent DNA strands in *FEN1*^*−/−*^ chicken DT40 cells. **a**, Schematic for measuring the size distribution of nascent DNA strands in WT and *FEN1*^*−/−*^ DT40 cells following 10 min of pulse labeling with ^3^H-thymidine (^3^H-THM) and during a subsequent 5 and 20 min of chase with thymidine (THM), in the absence or presence of PARPi (10 µM Olaparib) as indicated, by alkaline agarose gel electrophoresis. **b**, Quantification of ^3^H-pulse-labeled nascent DNA strands in the size ranges <0.5, 0.5–10 and >10 kb by liquid scintillation counting of radioactivity in alkaline agarose gel slices. Graphs show the mean fraction (%) ±1 s.d. of ^3^H radioactivity (in counts per minute (CPM)) in nascent DNA fragments of the indicated sizes from three independent experiments. Statistical analysis of the mean fractions (±s.d.) of radioactivity detected as nascent DNA strands of <10 kb from three independent experiments (*n* = 3) was conducted by two-way ANOVA with Tukey’s post hoc multiple comparisons test (NS, not significant; *P < 0.05; **P < 001). Source data

BrdU comet assays are sensitive only to nascent DNA fragments of 500 kilobases (kb) or more (Extended Data Fig. 2e), and so cannot measure the integrity of early DNA replication intermediates such as newly formed Okazaki fragments. To do this, we used alkaline agarose gels, in which the distribution of nascent DNA single strands of <10 kb can be resolved (Fig. 3a). An increased fraction of nascent DNA was present as fragments of 10 kb or less in *FEN1*^*−/−*^ DT40 cells, when compared to WT cells, following a 10-min pulse label with [^3^H]-thymidine, and remained so throughout a subsequent 20-min chase (Fig. 3b). Moreover, although PARP inhibitor did not measurably affect the amount of nascent DNA present as fragments of <10 kb in WT DT40 cells, it had a significant impact on the amount of these fragments in *FEN1*^*−/−*^ cells, increasing their prevalence during both pulse labeling and the subsequent chase (Fig. 3b). Collectively, these data indicate that PARP inhibitor impedes the maturation of nascent replication intermediates in DT40 cells, and that this effect is particularly pronounced if canonical Okazaki fragment processing is perturbed.

### PARP inhibition and postreplicative nascent single-strand gaps

**Fig. 4:**
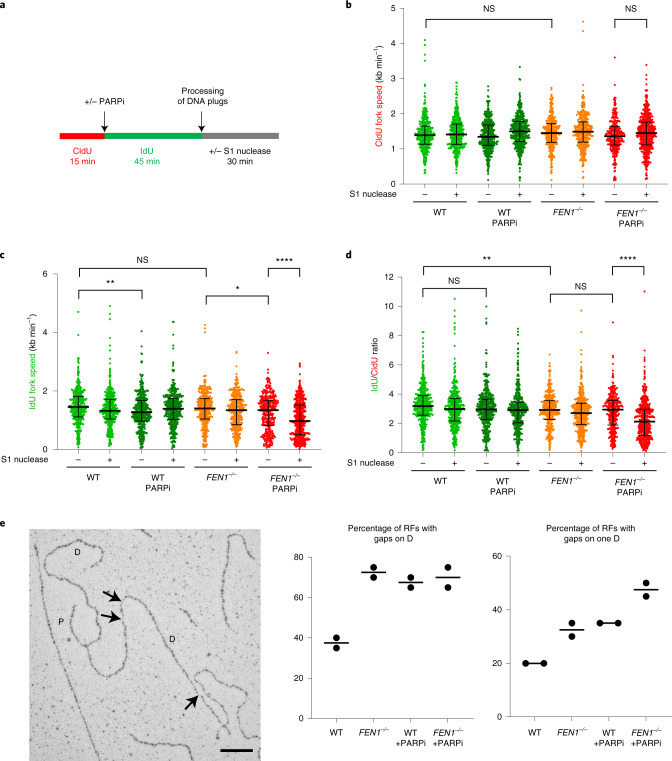
PARP inhibitor induces postreplicative single-strand gaps in *FEN1*^*−/−*^ DT40 cells. **a**, Schematic for measuring the rates of DNA replication fork progression during consecutive CldU and IdU pulse labeling by DNA combing in WT and *FEN1*^*−/−*^ DT40 cells incubated or not in PARPi (10 µM Olaparib) during the IdU pulse label. **b**, CldU tract lengths in dual-labeled DNA fibers in the indicated cell lines, plotted as scatter plots of fork speed combined from *n* = 3 independent experiments (>65 fibers per sample per experiment). Bars represent the median and interquartile range (see Extended Data Fig. 3a for individual data sets). Statistical analysis was conducted by two-way ANOVA with Tukey’s post hoc multiple comparisons test. Relevant comparisons are shown (NS, not significant; *P < 0.05; **P < 0.01; ****P < 0.0001). **c**, IdU tract lengths in dual-labeled DNA fibers scored in *n* = 3 independent experiments. Data are plotted and statistical analysis is as in **b** (see Extended Data Fig. 3b for individual data sets). **d**, The ratio of IdU and CldU tract lengths in individual dual-labeled fibers from the data in **b** and **c**, plotted as scatter plots and statistical analysis as in **b** (see Extended Data Fig. 3c for individual data sets). **e**, Representative electron microscopic image (left) and quantification (right) of replication forks with daughter strand single-strand gaps (arrows), following treatment of WT and *FEN1*^*−/−*^ DT40 cells with DMSO vehicle or 10 µM PARPi (KU0058948) for 1 h before analysis. P, parental strand and D, daughter strand. Scale bar, 100 nm. Graphs show the fraction of forks with detectable gaps on at least one daughter strand (left graph) or on a single daughter strand (right graph) in two independent experiments (20 forks were scored per sample, per experiment). The image is from *FEN1*^*−/−*^ cells treated with PARPi. Source data

**Extended Data Fig. 3:**
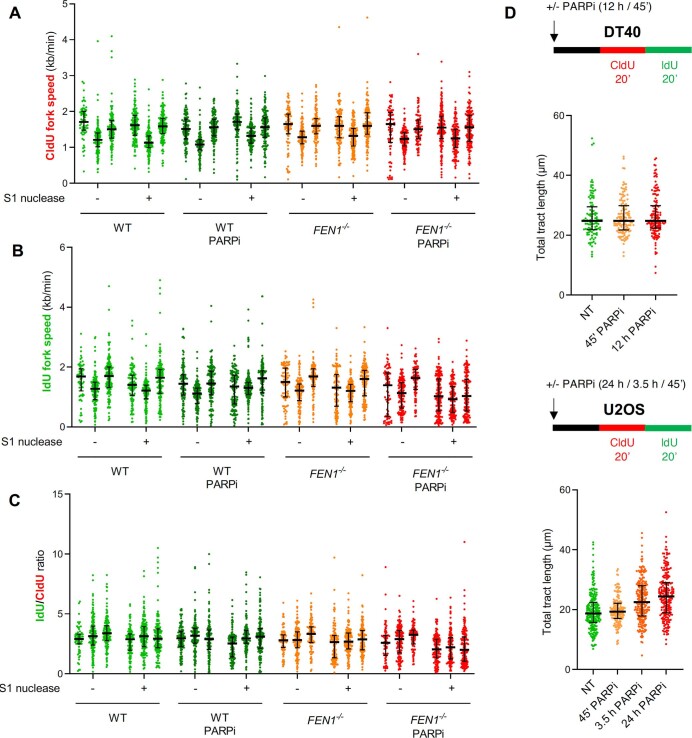
PARP inhibitor induces post-replicative single-strand gaps in *FEN1*^*-/-*^ DT40 cells. **(a-c)** Scatter plots of the individual experimental data sets (n = 3) that comprise Fig. 4b–d, respectively, with each data point plotted separately and experimental repeats plotted side by side. Bars represent the median and interquartile range. **(d)** Lengths of DNA replication tracts measured by CldU/IdU pulse labeling and DNA combing in wild type DT40 (*top*) and U2OS (*bottom*) cells incubated or not with PARPi (10 µM Olaparib) for 45 min, 3.5 h, or 24 h, as indicated. Total tract-lengths (CldU + IdU) of dual-labelled DNA fibres from n = 1 experiment (>100 fibres per sample) are presented, as scatter plots. Bars depict the median and interquartile range. Source data

To confirm that the impact of PARP inhibitor on nascent strand integrity reflected the induction and/or persistence of postreplicative nicks and/or gaps, we used DNA combing. DT40 cells were labeled for 15 min with 5-chloro-2′-deoxyuridine (CldU) followed by a further 45 min of labeling with 5-iodo-2′-deoxyuridine (IdU) in the presence or absence of PARP inhibitor, and the length of individual DNA replication tracts was quantified (Fig. 4a). Where indicated, genomic DNA was treated with S1 nuclease before DNA combing to detect postreplicative single-strand nicks and gaps. DNA replication fork rates were similar in WT and *FEN1*^*−/−*^ DT40 cells and were affected only slightly by PARP inhibitor over the time course of the experiments (Fig. 4b–d and Extended Data Fig. 3a–c; ‘-S1 nuclease’ samples), suggesting that PARP inhibition did not greatly affect the frequency and/or persistence of fork stalling, collapse or reversal in these cells. We did, however, detect the previously reported increase in fork rates in human U2OS cells incubated for prolonged periods (24 h) with PARP inhibitor^45^, suggesting that the impact of PARP inhibitor on fork progression is time and/or cell type dependent (Extended Data Fig. 3d). More importantly, S1 nuclease reduced the median length of IdU replication tracts synthesized in the presence of PARP inhibitor by roughly 30% in *FEN1*^*−/−*^ DT40 cells, confirming that PARP inhibition markedly increased the presence of postreplicative single-strand nicks/gaps located tens-of-kb behind DNA replication forks, if canonical Okazaki fragment processing was perturbed (Fig. 4b–d and Extended Data Fig. 3a–c). Finally, in an attempt to capture and visualize directly single-strand gaps located very near (<2 kb) to DNA replication forks, and thus very close to their time of origin, we used electron microscopy. These experiments confirmed the increased presence of single-strand gaps located behind DNA replication forks in DT40 cells lacking FEN1 and/or treated with PARP inhibitor (Fig. 4e).

### PARP inhibitor and nascent strand integrity in human cells

**Fig. 5:**
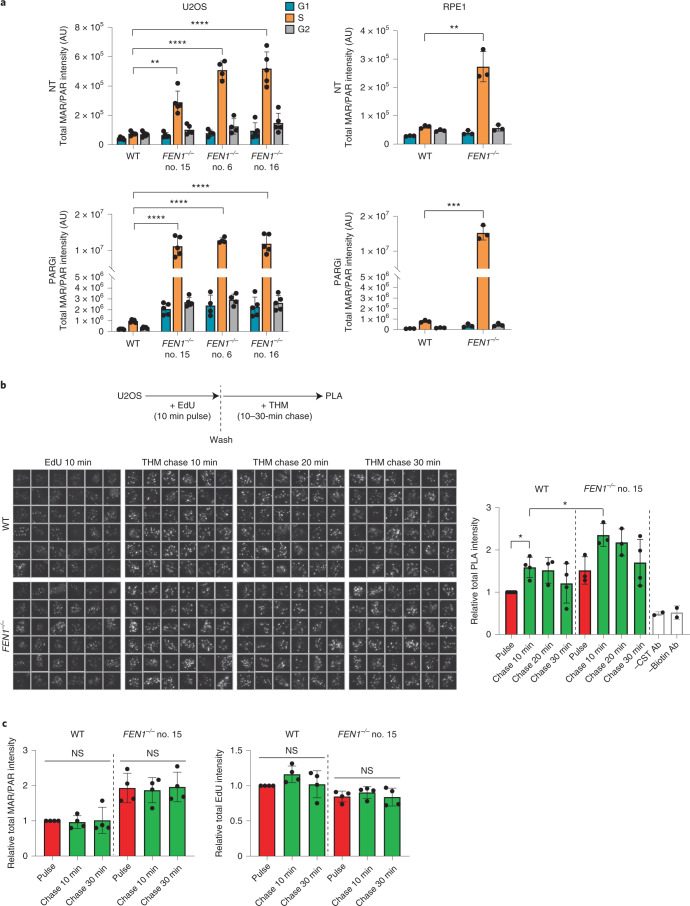
S phase PARP activity is highest behind DNA replication forks in human cells. **a**, ScanR quantification of anti-ADP-ribose immunofluorescence (detected using CST 83732) in WT and *FEN1*^*−/−*^ U2OS cells (clone nos. 15, 6, 16, left) and RPE-1 cells (right), incubated with or without (NT) PARG inhibitor (10 µM) for 30 min as indicated in detergent-extracted cells (see Extended Data Fig. 4b for representative images). Cell cycle phase was distinguished by PCNA staining and DNA content (DAPI staining). Data are the mean (±s.d.) total intensity of ADP-ribose in arbitrary units (AU) from *n* = 5 (left) or *n* = 3 (right) independent experiments (individual data points are also shown). Statistical significance was assessed by one-way ANOVA with post hoc Sidak’s multiple comparisons test (NS, not significant, *P < 0.05, **P < 0.01, ***P < 0.001, ****P < 0.0001). **b**, Physical proximity of newly incorporated EdU and ADP-ribose in WT and *FEN1*^*−/−*^ U2OS cells, measured by PLA following pulse labeling for 10 min and during a subsequent 30 min of thymidine (THM) chase (see schematic, top). PLA was measured following detergent extraction and paraformaldehyde fixation using antibiotin antibodies to detect biotin-azide clicked EdU and anti-ADP-ribose antibodies (CST 83732) to detect sites of PARP activity. Representative scanR image galleries (left, each box is a single cell) and quantification (right) are shown. Data are the mean PLA fluorescence signals (±s.d.) in PCNA-positive cells, normalized to that in WT U2OS cells immediately after EdU pulse labeling, from *n* = 2–4 independent experiments (individual data points are also shown). Statistics as in **a**, with only significant differences shown. **c**, Quantification of total ADP-ribose and EdU levels following pulse labeling and during the thymidine chase in the experiments shown in **b**. Data are the mean fluorescence from *n* = 4 experiments (±s.d.) normalized, plotted and statistics as in **b**. Source data

**Extended Data Fig. 4:**
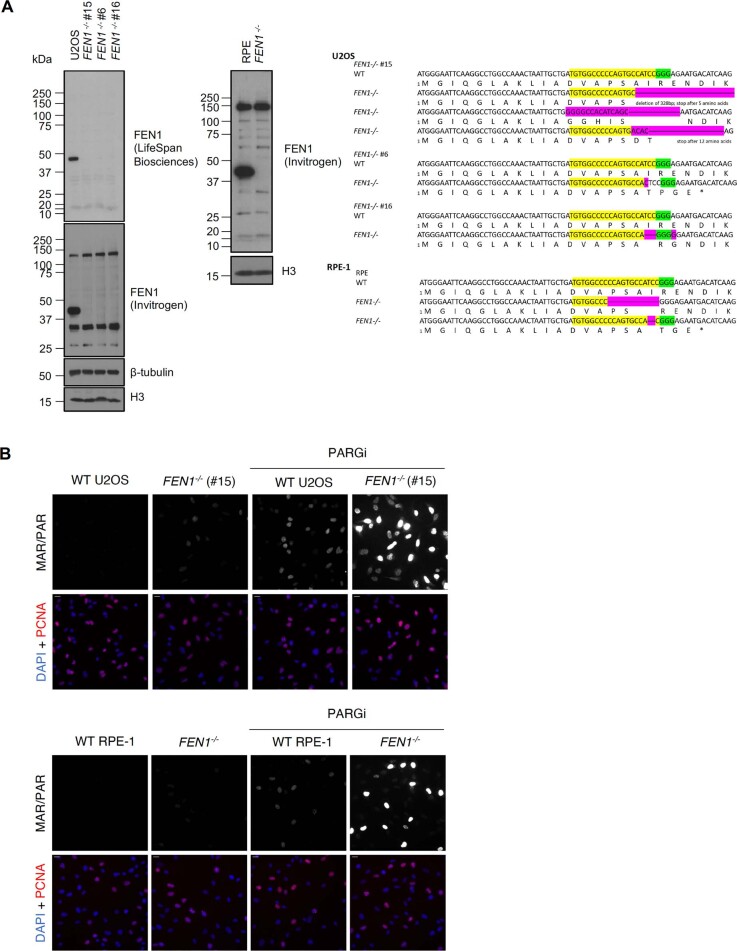
Generation of *FEN1*^*-/-*^ U2OS cells by CRISPR-Cas9 gene editing. **(a)** Analysis of WT U2OS/RPE-1 cells and the indicated *FEN1*^*-/-*^ clones by western blotting of whole cell extracts (*left*) and by Sanger sequencing (*right*) of PCR products of genomic DNA spanning the predicted Cas9 break site (n = 1). The PAM is shown in green, gRNA targeted sequence is shown in yellow and mutated nucleotides are shown in pink. **(b)** Representative scanR images of data quantified in Fig. 5a of anti-ADP-ribose immunofluorescence (CST 83732) in WT and *FEN1*^*-/-*^ (clone #15 only) U2OS cells (*top*), and in WT and *FEN1*^*-/-*^ RPE-1 cells (*bottom*), treated or not as indicated with 10μM PARG inhibitor (PARGi) for 30 min. Scale bars 20 μm. Source data

Collectively, our experiments with DT40 cells suggest that PARP1 is activated by unligated Okazaki fragments and that PARP inhibition impedes the maturation or repair of these structures over a wide range of distances behind DNA replication forks. To examine whether this is also the case in human cells, we disrupted FEN1 in U2OS and RPE-1 cells by gene editing (Extended Data Fig. 4a). Similar to DT40 cells, *FEN1*^*−/−*^ U2OS and RPE-1 cells exhibited higher levels of ADP ribosylation in S phase than did WT cells, and did so even in the absence of PARG inhibitor (Fig. 5a and Extended Data Fig. 4b). To confirm that this activity was located behind DNA replication forks, we compared the proximity of ADP-ribose and 5-ethyl-2′-deoxyuridine (EdU)-labeled tracts of nascent DNA immediately after pulse labeling and following a subsequent thymidine chase, by proximity ligation assays (PLA). While we detected significant PLA signal in WT U2OS cells immediately after pulse labeling for 10 min, this signal increased significantly during a subsequent 10-min chase (Fig. 5b). A similar trend was observed in *FEN1*^*−/−*^ U2OS cells, but as expected with overall higher levels of PLA signal (Fig. 5b). The increase in PLA signal in U2OS cells during a 10-min chase did not reflect a general increase in either EdU or ADP-ribose, because this was similar throughout the experiment (Fig. 5c). Together, these data indicate that levels of S phase ADP ribosylation are highest behind DNA replication forks.

**Fig. 6:**
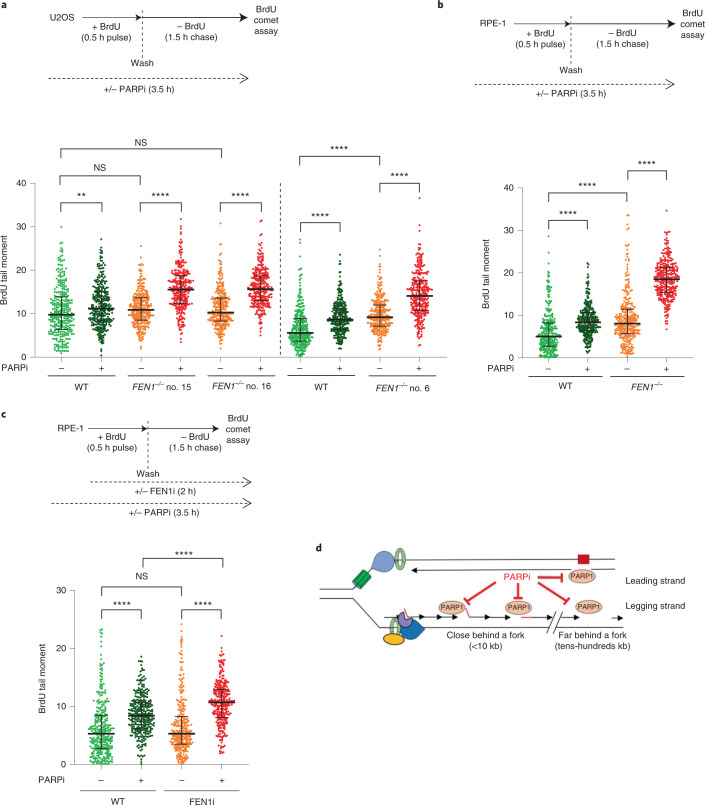
PARP inhibitor impedes the maturation of nascent DNA strands in human cells. **a**, DNA breaks quantified in nascent DNA strands of the indicated U2OS cells by alkaline comet assays following pulse labeling (0.5 h) with BrdU and a subsequent chase (1.5 h) as indicated (see schematic, top). 10 µM PARPi (Olaparib) was used as indicated. DNA breaks in nascent DNA strands were scored by staining with anti-BrdU antibodies. Clone no. 6 and clone nos. 15/16 were analyzed and quantified in different experimental sets. For each sample, scatter plots show BrdU tail moments from 300 cells combined from *n* = 3 experiments (100 cells per sample per experiment) and bars are the median and interquartile range (see Extended Data Fig. 5b for individual data sets). Statistical significance was determined by two-way ANOVA with Tukey’s post hoc tests (NS, not significant, **P < 0.01, ****P < 0.0001). **b**, DNA breaks quantified in nascent DNA strands of the indicated RPE-1 cells (*n* = 3), as described in **a** (see Extended Data Fig. 5c for individual data sets). Statistics as in **a**, above. **c**, DNA breaks quantified in nascent DNA strands in WT RPE-1 cells by alkaline comet assays (*n* = 3), as described in **a**. Where indicated (see schematic, top), cells were incubated with 10 µM PARPi (Olaparib) and/or 10 µM FEN1 inhibitor (FEN1i). Separate experimental repeats plotted in Extended Data Fig. 5d. Statistics as in **a**, above. **d**, A model for PARP1 trapping on nascent strand discontinuities during DNA replication. While most Okazaki fragments are processed and ligated very rapidly some escape processing by the canonical pathway(s) (for example, using FEN1/LIG1) and are detected by PARP1 over a broad range of distances behind the fork (from several kb to hundreds of kb). While we propose that these unligated Okazaki fragments are the major source of PARP1 activity during S phase, we do not exclude activation at other nascent (or leading) strand discontinuities, such as those created by repriming and lesion bypass (red box). Source data

**Extended Data Fig. 5:**
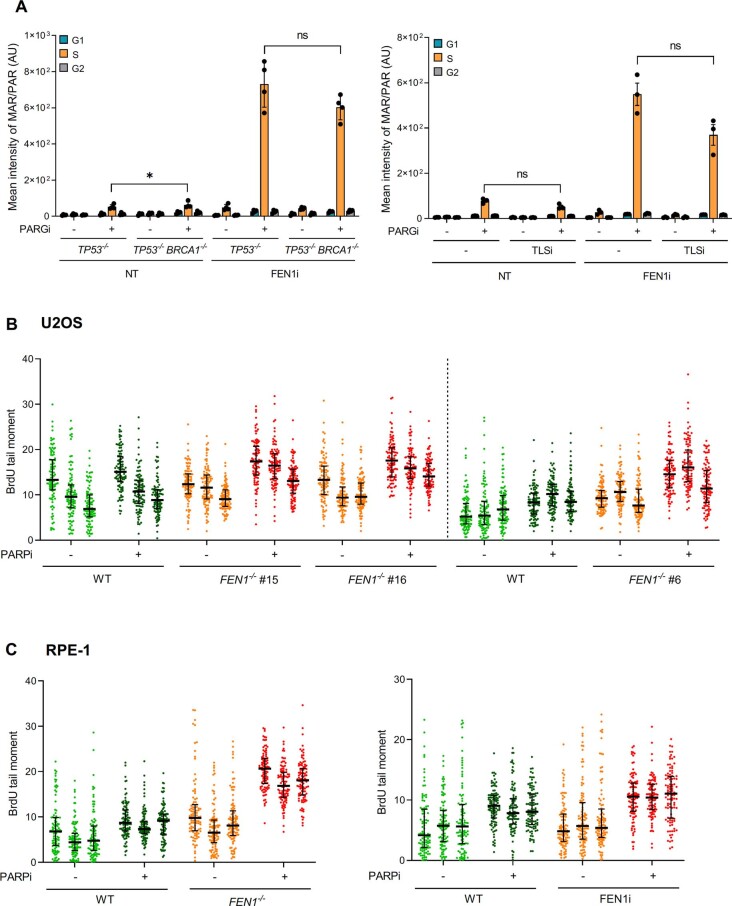
S phase ADP-ribose levels and nascent strand integrity in human FEN1-defective cells. **(a)** ScanR quantification of ADP-ribose (MAR/PAR, detected by CST 83732) in *TP53*^*-/-*^ and *TP53*^*-/-*^ *BRCA1*^*-/-*^ RPE-1 cells (*left)*, and in WT RPE-1 cells treated or not for 2 h with 20 µM of the REV1 inhibitor JH-RE-06 (TLSi; *right*), co-incubated or not (NT) as indicated for 90 min with 20 µM FEN1 inhibitor (FEN1i). Where indicated, 10 µM PARG inhibitor (PARGi) was present during the last 30 min to prevent poly(ADP-ribose) degradation. Cell cycle phase was determined by PCNA staining and DNA content (DAPI intensity). Data are the mean (±SD) ADP-ribose fluorescence in arbitrary units (AU) from three (left) or four (right) independent experiments with individual data points also plotted. Statistical significance was assessed by 1-way ANOVA with post-hoc Sidak’s multiple comparisons test (ns, not significant; *p < 0.05). **(b-d)** Scatter plots of the individual experimental data sets (n = 3) that comprise Fig. 6a–c, respectively, with each data point plotted separately and experimental repeats plotted side by side. Bars represent the median and interquartile range. Source data

**Extended Data Fig. 6:**
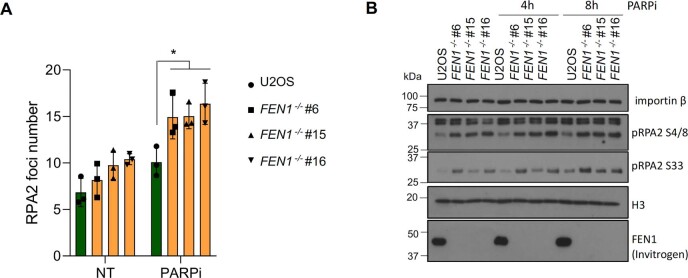
Impact of PARP inhibitor and FEN1 deletion on RPA foci and phosphorylation. **(a)** ScanR quantification of RPA2 foci in wild type U2OS cells and the indicated *FEN1*^*-/-*^ clones treated or not (NT) for 8 h with 10 µM PARP inhibitor (PARPi). S phase cells were distinguished by PCNA staining and DAPI intensity. Data represent the average (±1 SD) RPA2 foci number from three independent experiments with individual data points plotted. Statistical significance was assessed by 1-way ANOVA with post-hoc Sidak’s multiple comparisons test. **(b)** Western blotting analysis of whole cell extracts from wild type U2OS cells and the indicated *FEN1*^*-/-*^ clones treated or not (NT) for 4–8 h with 10 µM PARP inhibitor (PARPi)(n = 2). Source data

Since PARP activity behind DNA replication forks may be triggered not only by unligated Okazaki fragments but also by single-strand gap intermediates of translesion synthesis (TLS) and/or HR-mediated repair, we examined the impact of these pathways on S phase PARP activity in human cells. However, neither a REV1/TLS inhibitor (JH-RE-06)^46^ nor deletion of the HR protein BRCA1 (ref. ^35^) greatly affected levels of S phase ADP ribosylation, suggesting that these pathways are not major contributors in S phase PARP activity, even in the presence of FEN1 inhibitor (Extended Data Fig. 5a). To confirm that PARP inhibitors impede the repair and/or maturation of nascent DNA strands in human cells, we used alkaline BrdU comet assays. Similar to DT40 cells, incubation with PARP inhibitor reduced the integrity of nascent DNA strands in WT U2OS and RPE-1 cells during DNA replication, and did so to a greater extent in *FEN1*^*−/−*^ cells (Fig. 6a,b and Extended Data Fig. 5b,c). Similar results were observed in human RPE-1 cells in which we perturbed FEN1 activity by chemical inhibition (Fig. 6c and Extended Data Fig. 5c). Finally, levels of RPA2 foci and RPA2 phosphorylation were elevated in *FEN1*^*−/−*^ U2OS cells during S phase, and were further increased by PARP inhibitor, consistent with the presence of increased single-strand gaps (Extended Data Fig. 6a,b).

In summary, we show here that PARP activity is greatest behind DNA replication forks and that PARP inhibitors impede the maturation of nascent DNA strands during DNA replication. Moreover, the impact of PARP inhibition on nascent strand integrity is particularly pronounced in cells lacking FEN1 activity, supporting the idea that unligated Okazaki fragments are an endogenous source of PARP inhibitor-induced genotoxicity.

## Discussion

PARP inhibitors provide a powerful new approach in the treatment of cancer, particularly in tumor cells in which HR is attenuated or absent^30–32^. By trapping PARP1 on DNA lesions PARP inhibitors render proliferating cells dependent on HR for cell survival. However, the mechanisms by which this trapping affects DNA metabolism and exerts cytotoxicity have been unclear^10^. Here, we have found that PARP inhibitors decrease the integrity of nascent DNA strands during DNA replication. It is unlikely that this finding is explained by an impact of PARP inhibitor on DNA replication fork progression, resulting from the role identified for PARP1 in regulating replication fork reversal and/or repair following treatment of cells with genotoxins^18,19,47^. This is because PARP inhibitor did not greatly affect DNA replication fork rates in DT40 cells in our experiments, as measured by DNA combing, suggesting that the requirement for PARP1 to regulate replication fork progression was too rare to be detected in these cells.

In contrast to the lack of impact on DNA replication fork rates, our DNA combing experiments detect an increased number of postreplicative single-strand gaps in *FEN1*^*−/−*^ cells following treatment with PARP inhibitor. This is in agreement with the greatly exacerbated impact of PARP inhibitor on nascent strand integrity detected in these mutant cells by alkaline comet assays and alkaline gel electrophoresis. FEN1 has multiple roles in DNA metabolism, including during DNA BER and RER pathways that are operative during S phase^43,44^. However, these roles are unlikely to account for the elevated impact of PARP1 inhibitor on nascent strand integrity in FEN1-defective cells, because deletion of the APE1 and Rnaseh2 enzymes that create DNA strand breaks during these pathways did not affect the level of S phase PARP activity induced by FEN1 inhibitor. Similarly, we did not detect a significant impact on the level of S phase PARP activity induced by FEN1 inhibitor if we also inhibited TLS or BRCA1-dependent DNA processing, both of which are important for DNA replication at sites of DNA lesions^48,49^. Thus, while we do not exclude a contribution from other DNA lesions and/or DNA structures, our results best fit a model in which the most common source of S phase PARP1 activity and trapping are unligated Okazaki fragments.

Our experiments suggest that PARP1 inhibitor affects the integrity of nascent DNA over a wide range of distances behind DNA replication forks, from within several to hundreds of kb. While we detected an effect of PARP inhibitor on the integrity of large nascent strands of >500 kb in both WT and *FEN1*^*−/−*^ cells, the impact of PARP inhibitor on smaller nascent fragments of <50 kb was only evident in *FEN1*^*−/−*^ cells. We suggest that this reflects the lower sensitivity of the alkaline gel electrophoresis and fiber assays used to measure the smaller nascent DNA fragments. For example, whereas the number of nascent DNA strand breaks remaining unrepaired in the presence of PARP inhibitor in these experiments was enough to measurably affect the size distribution of the large (>500 kb) nascent fragments, it may have been too small to measurably affect the size distribution of smaller (<50 kb) nascent fragments, unless FEN1 was also inhibited. We did detect an impact of PARP inhibitor in WT cells on the number of single-strand gaps located very close (<2 kb) to DNA replication forks by electron microscopy. While such gaps were not necessarily located on nascent strands it seems likely that many were, given the impact of FEN1 deletion on their number. Based on a replication fork speed of around 1 kb min^−1^ these gaps were detected within roughly 2 min of their generation, most likely explaining why more were detectable.

Collectively, our data indicate that PARP1 detects incompletely processed Okazaki fragments that have escaped the canonical pathway, some of which persist for large distances behind the departing replisome (Fig. 6d). The idea that PARP activity is greatest behind DNA replication forks is consistent with our PLA data, which revealed that sites of EdU-labeled nascent DNA were nearer to sites of ADP ribosylation following a 10-min chase than immediately after a 10-min pulse label. The PLA signal appeared to decline thereafter in our experiments, perhaps reflecting the dissociation of PARP1 from the pulse-labeled nascent DNA following SSB/gap repair.

The cytotoxicity of PARP inhibitors reflects, in part at least, the trapping of PARP1 on DNA breaks, which impedes their repair by other DNA repair enzymes^32,33^. Our data indicate that unligated Okazaki fragments comprise a significant fraction of the DNA structures on which PARP1 is trapped by PARP inhibitors, in S phase. Indeed, given the hypersensitivity of *FEN1*^*−/−*^ DT40 cells to PARP inhibitor^33^, which in our experiments was greater than that of HR-deficient *XRCC3−*^*/*^*−* cells, unligated Okazaki fragments may be a major source of PARP1 trapping and cytotoxicity in proliferating cells. This idea is consistent with recent reports that single-strand gaps are a major source of the hypersensitivity induced in BRCA1/BRCA2 mutated cells^38,50,51^. Whether PARP1 plays an active role in processing Okazaki fragment intermediates or is simply trapped on these structures by PARP inhibitor remains to be determined, although the observation that SSB repair proteins such as XRCC1 and LIG3 are recruited to sites of S phase PARP1 activity and can replace LIG1 during Okazaki fragment processing is consistent with the former^11,27,28,42^. Nevertheless, our results implicate Okazaki fragments and likely other nascent strand discontinuities as major sources of genome breakage and cytotoxicity during treatment with PARP inhibitors. Our data thus shed light on the source of DNA breaks that might underpin the clinical use of PARP inhibitors in cancer therapy.

## Methods

### Chemicals

The chemicals used were 100 mM stock solution of BrdU (Merck, B5002) and 10 mM stock solutions of PARG inhibitor (PDD0017273, Tocris, 5952; Merck, SML1781), PARP inhibitors (KU0058948, Axon Medchem, Axon 2001; Olaparib, ApexBio, A4154), FEN1 inhibitor (UOS-33991; compound 17 in ref. ^52^ and synthesized in-house as described in ref. ^11^), REV1 TLS inhibitor (JH-RE-06, Axon Medchem, Axon 3002) and EdU (Cambridge Bioscience, CAY20518) were prepared in dimethyl sulfoxide (DMSO). CldU (Merck, C6891) and IdU (Merck, I7125) were dissolved directly in culture medium at a final concentration of 2.5 mM, and thymidine (Merck, T1895) in culture medium at 200 mM. 1 mCi per ml ^3^H-Thymidine (PerkinElmer, NET027W005MC) in 2% ethanol and 99% MMS were added directly to culture medium to a final concentration of 2 µCi per ml and 0.01%, respectively.

### Antibodies

Primary antibodies used were: anti-poly-ADP-ribose (PAR) binding reagent (Millipore, MABE1031; 1:200–1:500), rabbit anti-mono/poly-ADP-ribose Mab (PAR/MAR; Cell Signaling, 83732; 1:500), mouse anti-PCNA Mab (Santa Cruz, sc-56; 1:500), rabbit anti-PARP1 Mab (Cell Signaling, 9532; 1:2,000), rat anti-α-tubulin polyclonal (Abcam, ab6160; 1:5,000), rabbit anti-H3 polyclonal (Abcam, ab1791; 1:5,000), rabbit anti-FEN1 polyclonal (LifeSpan Biosciences, LS-C80825 1:500), mouse anti-FEN1 Mab (Invitrogen, MA1-23228, 1:1,000), mouse antibiotin Mab (Merck, BN-34, 1:100), rat recombinant anti-PCNA (Abcam, ab252848, 1:2,000), rat anti-BrdU Mab (Abcam, ab6326 1:50), mouse anti-BrdU Mab (Becton Dickinson, 347580; 1:2–1:25), mouse anti-single-strand DNA Mab (Millipore, MAB3034; 1:25), mouse anti-RPA2 Mab (Abcam, ab2175, 1:200), rabbit anti-RPA2 pS33 polyclonal (NB100-544, Novus Biologicals, 1:5,000), rabbit anti-RPA2 pS4/8 polyclonal (Millipore, PLA0071, 1:4,000), mouse anti-importin β Mab (Santa Cruz, sc-137016, 1:1,000). Secondary antibodies used were: HRP-conjugated goat anti-rabbit (Dako, P0448; 1:5,000), HRP-conjugated goat anti-mouse (Bio-Rad, 170-6516; 1:5,000), HRP-conjugated rabbit anti-mouse (Dako, P0260; 1:5,000), rabbit anti-rat (Abcam, ab6734; 1:5,000), donkey anti-rabbit Alexa Fluor 488 (Thermo Fisher, A21206; 1:500–1:1,000), donkey anti-mouse Alexa Fluor 568 (Thermo Fisher, A10037; 1:500–1:1,000), donkey anti-mouse Alexa Fluor 647 (Thermo Fisher, A31571 1:25–1:1,000), goat anti-mouse Alexa Fluor 488 (Thermo Fisher, A11001; 1:1,000), goat anti-mouse Alexa Fluor 488 (Thermo Fisher, A32723; 1:25–1:1,000), donkey anti-goat Alexa Fluor 488 (Thermo Fisher, A11055; 1:250), goat anti-rat Alexa Fluor 568 (Thermo Fisher, A11077; 1:25) and donkey anti-rat Alexa Fluor 488 (Thermo Fisher, A21208, 1:500).

### Cell culture

Human WT (ATCC, CRL-4000), *TP53*^*−/−*^ (ref. ^35^), *TP53*^*−/−*^*/BRCA1*^*−/−*^ (ref. ^35^), *FEN1*^*−/−*^, *XRCC1*^*−/−*^ (ref. ^53^) and *XRCC1*^*−/−*^*/APE1*^*−/−*^ hTERT RPE-1 cells were maintained in Dulbecco’s Modified Eagle’s Medium (DMEM/F12, Merck) supplemented with 10% fetal calf serum (FCS). Human WT (ATCC, HTB-96) and *FEN1*^*−/−*^ U2OS cells, and *Rnaseh2b*^*−/−*^ mouse embryonic fibroblasts^54^ were cultured in DMEM (Gibco) with 10% FCS and 2 mM l-glutamine (Gibco). All above cells were grown under 3% oxygen. The generation of *FEN1*^*−/−*^ U2OS and RPE-1 cells is described below, and the generation of *APE1*^*−/−*^ cells will be described in detail elsewhere. Chicken WT and *FEN1*^*−/*−^ (ref. ^29^) DT40 cell lines were cultured in RPMI 1640 medium supplemented with 10% FCS, 1% chicken serum (Gibco), 2 mM l-glutamine and 10 µM β-mercaptoethanol (Gibco). All growth media was supplemented with penicillin (100 units per ml)/streptomycin (100 mg ml^−1^) (Merck) and all cells were grown at 37 °C.

### Purification of SpCas9 and generation of *FEN1*^*−/−*^ U2OS and RPE-1 cells

His-SpCas9-green fluorescent protein (GFP) was expressed in and purified from BL21 (DE3, NEB, C2527H) bacteria as previously described^55^. Briefly, inoculated culture was grown to an optical density (OD_600_) of 0.5, cooled to 16 °C and Cas9 expression induced with 0.1 mM IPTG for 20 h. Cells were resuspended in lysis buffer (50 mM Tris pH 7.5, 500 mM NaCl, 20 mM imidazole, 1 mM TCEP) supplemented with protease inhibitors, sonicated and centrifuged at 20,000*g* for 40 min at 4 °C. The supernatant was incubated with Ni-NTA agarose beads (GE Healthcare, 17-5318-01) for 1 h at 4 °C, the beads were extensively washed with lysis buffer, followed by lysis buffer containing 150 mM NaCl. His-SpCas9-GFP was eluted with 300 mM imidazole in 50 mM Tris pH 7.5, 150 mM NaCl, 1 mM TCEP, diluted with 25 mM HEPES pH 7.4, 150 mM NaCl, 1 mM TCEP and loaded onto a 5-ml HiTrap SP HP column (GE Healthcare, 17-1152-01). After extensive washing, protein was eluted with a linear gradient to 0.6 M NaCl over 25 column volumes (CV), followed by 8 CV to 1 M NaCl. Then fractions of 2.5 ml were collected and snap frozen in liquid nitrogen before use. To generate Cas9 RNPs for electroporation, 120 pmol crispr RNA (Merck, UGUGGCCCCCAGUGCCAUCC) was mixed with 120 pmol trans-activating crispr RNA (tracrRNA) (Merck, TRACRRNA05N) in 1:1 molar ratio in Cas9 buffer (20 mM HEPES pH 7.5, 150 mM NaCl, 2 mM MgCl_2_, 1 mM TCEP) before addition of 100 pmol His-Cas9-GFP and incubation for 10 min at room temperature. 2 × 10^5^ U2OS cells were washed in PBS and electroporated using Neon transfection system (Thermo Fisher) with a 10-µl tip using 1,230 V per 10 width per 4 pulses (for U2OS) or 1,350 V per 20 width per 2 pulses (for RPE-1). After 3 d, cells were reseeded in a 96-well plate at 0.5 cells per well. Single cell clones were amplified and analyzed by western blotting and genomic DNA was isolated (DNeasy Blood and Tissue Kit, Qiagen, 69504). The locus surrounding the Cas9 cutting site was amplified using Q5 DNA polymerase (NEB, M0491S) and primers flanking the Cas9 cut site,

FWD: TGGTGCCGCGCGGCAGCCACCTGTCTTTCAGGTCTGCCAT,

REV: CACCAGTCATGCTAGCCATATTCACTGGCAGTCAGGTGTC.

PCR products were purified (QIAquick PCR Purification Kit, Qiagen, 28106), cloned into *Nde*I-cut pET28a using NEBuilder HiFi DNA Assembly Master Mix (NEB, E2621S) and plasmid DNA from single colonies purified and Sanger sequenced.

### PLA

Cells were seeded at 2 × 10^5^ per well in a six-well plate and next day incubated with 100 µM EdU (Cambridge Bioscience, CAY20518) for 10 min, rinsed (3×) and incubated as indicated in media containing 100 µM thymidine (Merck). Before fixation, cells were washed with PBS, pre-extracted using pre-extraction buffer (25 mM HEPES pH 7.4, 50 mM NaCl, 1 mM EDTA, 3 mM MgCl_2_, 0.3 M sucrose, 0.5% Triton X-100) supplemented with 10 µM PARPi (KU0058948, Axon Medchem, Axon 2001) and PARGi (PDD0017273, Merck, SML1781) for 5 min on ice, and fixed with cold 4% formaldehyde for 15 min. Cells were permeabilized using ice-cold methanol/acetone solution (1:1) for 5 min and PBS containing 0.5% Triton X-100 and blocked in bovine serum albumin (BSA). The click reaction was performed using 0.1 M Tris pH 8.5, 0.1 M sodium ascorbate, 2 mM Cu_2_SO_4_ and 0.1 mM biotin-azide (Merck, 762024) or Alexa Fluor 647 azide (Thermo Fisher, A10277) for 45 min at room temperature. After washing, cells were stained with the indicated primary antibodies for 2 h at room temperature followed by incubation with PLA probes (Merck, Duolink In Situ PLA Probe Anti-Rabbit PLUS, DUO92002, Duolink In Situ PLA Probe Anti-Mouse MINUS, DUO92004) for 1 h at 37 °C, ligation for 30 min 37 °C, and polymerase reaction overnight at 37 °C according to the manufacturer’s protocol (Merck, Duolink In Situ Detection Reagents Red, DUO92008). Images were acquired using an Olympus IX81 microscope equipped with a scanR screening system using a ×40 objective at a single autofocus-directed *z*-position under nonsaturating settings. Olympus scanR image analysis software was used to analyze and quantify the fluorescence intensity of PCNA-positive cells (hundreds or thousands per sample). Nuclei were identified by 4,6-diamidino-2-phenylindole (DAPI) signal using an integrated intensity-based object detection module.

### Indirect immunofluorescence

Human cells were seeded at 2 × 10^5^ per well in a six-well plate and the next day treated or not with 10 µM PARG inhibitor (PDD0017273, Merck, SML1781) for 30 min. Before fixation, cells were washed with PBS, incubated with pre-extraction buffer (25 mM HEPES pH 7.4, 50 mM NaCl, 1 mM EDTA, 3 mM MgCl_2_, 0.3 M sucrose, 0.5% Triton X-100) supplemented with 10 µM PARPi (KU0058948, Axon Medchem, Axon 2001) and PARGi (PDD0017273, Merck, SML1781) for 5 min on ice, and then fixed with cold 4% formaldehyde for 15 min. Cells were permeabilized using ice-cold methanol/acetone solution (1:1) for 5 min and PBS containing 0.5% Triton X-100 and blocked in BSA. Cells were stained with indicated primary antibodies for 2 h at room temperature followed by incubation with secondary antibodies for 1 h at room temperature and DNA counterstained with DAPI. DT40 cells were collected, washed and diluted in ice-cold PBS to a final concentration of roughly 7 × 10^5^ cells per ml. The cell suspension was centrifuged on a microscope slide (Thermo Fisher Scientific) (200 µl per slide) at 800*g* for 3 min in a Cytospin centrifuge, and PAP Pen Liquid Blocker (Merck) was used to draw a circle around a specimen to hold reagents within the area containing cells. Then, cells were fixed with 4% formaldehyde in PBS for 10 min at room temperature, rinsed in PBS, and then permeabilized with ice-cold methanol/acetone solution (1:1) for 5 min at room temperature, followed by three short washes in PBS. Next, cells were incubated in blocking solution (3% BSA in PBS) for 1 h at room temperature, followed by incubation with appropriate primary antibodies (1 h at room temperature) and then with fluorochrome-conjugated secondary antibodies (1 h at room temperature). Slides were washed (3×) in PBS after all antibody incubations, and DNA was counterstained with DAPI (1 ug ml^−1^ in water for 5 min at room temperature), before mounting in fluoroshield (Merck). When cells were labeled with EdU, click reaction was carried out after the blocking step using Click-iT EdU Alexa Fluor 647 Imaging Kit (Invitrogen, C10640) according to the manufacturer’s instructions. Immunofluorescence images were acquired using an Olympus IX81 microscope equipped with scanR screening system using ×40 objective at a single autofocus-directed *z*-position under nonsaturating settings. Olympus scanR image analysis software was used to analyze and quantify the fluorescence intensity of individual cells (hundreds/thousands per sample). Nuclei were identified by DAPI signal using an integrated intensity-based object detection module. The G1, S and G2 phase cells were gated based on PCNA and DAPI intensity. High-resolution images in Fig. 1a were acquired with an Apotome widefield microscope (Zeiss) using a ×63 oil objective.

### Chromatin fractionation assay

DT40 cells (roughly 5 × 10^6^ per sample) were collected and lysed for 20 min on ice in 200 µl of CSK buffer (25 mM HEPES pH 7.4, 150 mM NaCl, 0.3 M sucrose, 3 mM MgCl_2_, 1 mM EDTA, 0.5% Triton X-100) containing protease inhibitors (Roche) and phosphatase inhibitors (Merck), and 50 µl aliquots were collected as samples of the total cell lysates. Soluble and insoluble/chromatin-bound proteins were separated by centrifugation (5 min 20,000*g* at 4 °C) and supernatants collected (soluble fractions). Pellets (insoluble fraction) were washed twice in 1 ml of CSK buffer and were dissolved in 150 µl of 2× Laemmli sample buffer (chromatin fractions). The following steps were the same as for western blotting (below).

### Western blotting

Cells were lysed in 2× Laemmli buffer lacking reducing agent (100 mM Tris-HCl pH 6.8, 4% SDS, 20% glycerol) followed by heating (99^o^C) for 5 min and sonication. Protein was quantified using BCA assays (Thermo Fisher Scientific), DTT and bromophenol blue, then added to 0.1 M and 0.1%, respectively, and samples reheated for 10 min at 99 °C. Samples were resolved on Bis-Tris SDS–PAGE gels in MOPS buffer (pH 7.7, 100–150 V) and transferred to nitrocellulose membrane (Thermo Fisher Scientific). Membranes were blocked for 1 h in 1× TBS containing 0.1% Tween20 (TBST) and 5% milk, followed by incubation with appropriate primary antibodies either for 1 h at room temperature or overnight at 4 °C. Membranes were then incubated with horseradish peroxidase-conjugated secondary antibody for 1 h at room temperature. Membranes were washed (3 × 10 min) in TBST at room temperature after each antibody incubation. Enhanced chemiluminescence detection reagent (GE Healthcare or Thermo Fisher Scientific) was applied and immunoreactive proteins were visualized either using ImageQuant LAS 4000 machine (Raytek) or chemiluminescence film (Scientific Laboratory Supplies or GE Healthcare).

### Clonogenic survival assay

WT, *FEN1*^*−/−*^ and *XRCC3*^*−/−*^ DT40 cells were seeded in triplicate in six-well plates at 100, 500 or 2,500 cells per well depending on PARPi dose in 5 ml of medium supplemented with 1.5% methylcellulose (Merck) and the indicated concentrations of Olaparib. Cells were grown for 10–14 d at 37 °C and visible colonies counted. Survival (%) was defined as the average number of colonies on treated plates divided by the average number of colonies on untreated plates multiplied by 100.

### Alkaline comet assays

Alkaline comet assays were performed essentially as described^56^. For measuring DNA breaks in total genomic DNA, slides were stained with SYBR Green (Merck, 1:10,000) or with propidium iodide (Merck, 1:500), and with *p*-phenylenediamine dihydrochloride (Thermo Fisher Scientific, 41 µg ml^−1^) in PBS as an antifade. To detect S phase cells and to detect DNA breaks specifically in nascent strands cells were pulse labeled with 100 µM BrdU for 30–45 min as indicated, and then ether sampled immediately (to detect S phase cells) or incubated for a subsequent 90-min chase period (to measure breaks in nascent strands during the maturation of DNA replication intermediates). After the neutralization step, slides were washed (3 × 10 min) in PBS, followed by incubation with the mouse monoclonal anti-BrdU antibody (Becton Dickinson, 347580; 1:2) overnight at 4 °C in a humid chamber. Excess primary antibody was removed and slides were then incubated simultaneously with two different secondary antibodies diluted in PBS/0.1% Tween20/3% BSA for 1 h at room temperature to amplify the signal (goat anti-mouse Alexa Fluor 488 (Thermo Fisher, A11001; 1:250) and donkey anti-goat Alexa Fluor 488 (Thermo Fisher, A11055; 1:250)). Thereafter, slides were washed 3 × 10 min in PBS and counterstained with propidium iodide (Merck, 1:500) and *p*-phenylenediamine dihydrochloride (Thermo Fisher Scientific, 41 µg ml^−1^) in PBS. In all cases, the lysis buffer was pH 10.4. Comet tail moments were visualized using Nikon Eclipse 50i widefield microscope and scored with Comet Assay IV software (Perceptive Instruments) in SYBR Green (with GFP filter) or propidium iodide (with fluorescein isothiocyanate filter) labeled DNA for total genomic DNA breaks, and in anti-BrdU-stained DNA (with GFP filter) for DNA breaks in DNA nascent strands.

### Alkaline agarose gel electrophoresis

Analysis of nascent DNA fragments by alkaline agarose gel electrophoresis was conducted as described in ref. ^27^. DT40 cells (roughly 5 × 10^6^ sample) were pulse labeled with ^3^H-thymidine (2 µCi ml^−1^) for 10 min, followed by 5–20 min of chase in fresh medium containing 2 mM thymidine. Cells were collected, washed in ice-cold PBS and resuspended in 20 µl of Buffer A (10 mM Tris-HCl, pH 8.0; 50 mM NaCl; 0.1 M EDTA). Next, the cell suspension, prewarmed for 10 s at 50 °C, was gently mixed with 25 µl of molten 1.5% low-melting-point agarose and pipetted into a casting mold (Bio-Rad), which was placed on ice for 5 min to solidify the agarose. Subsequently, the agarose plugs were lysed in 1 ml of Buffer A containing 0.2 mg ml^−1^ proteinase K (Thermo Fisher Scientific) and 2% *N*-lauryl sarcosine (Merck) for 18 h at 50 °C, followed by washing in 5 ml of Buffer A for 1 h at room temperature. The agarose plugs were then loaded on the comb, embedded in 1% alkaline agarose gel (1% agarose, 50 mM NaOH, 1 mM EDTA in H_2_O) and the genomic DNA fractionated by electrophoresis under denaturing conditions (50 mM NaOH, 1 mM EDTA in H_2_O) for 7.5 h (2 V cm^−1^) at room temperature. Following electrophoresis, the gel was neutralized for 1 h at room temperature in 1 M Tris-HCl, pH 7.6/1.5 M NaCl and stained with SYBR Green (Merck) (1:10,000) to visualize DNA molecular mass markers (0.075 to 20 kb). For each sample lane, the gel was cut into 1-cm long slices that were placed in scintillation vials and soaked in 0.1 M HCl for 1 h. The HCl solution was then carefully removed and the gel slices melted in a microwave. 4 ml of aqueous scintillant was thoroughly mixed with the melted gel slices by vortexing and ^3^H quantified (counts per minute) in a scintillation counter. The radioactivity in agarose slices corresponding to fragment sizes of <0.5, 0.5–10 and >10 kb were combined and plotted as percentages of the total counts per minute in all gel slices of that sample.

### DNA combing

DT40 cells were labeled with 25 µM CldU for 15 min, followed by labeling with 250 µM ldU for 45 min in the presence or absence of the PARP inhibitor Olaparib (10 µM). Next, cells were washed (2×) and resuspended in ice-cold PBS at roughly 5 × 10^6^ cells per ml. Then 50 µl of cell suspension, prewarmed for 10 s at 50 °C, was gently mixed with an equal volume of molten 1.5% low-melting-point agarose and pipetted into a casting mold (Bio-Rad) on ice for 10 min. The solidified agarose plugs were incubated in round-bottom 10 ml tubes containing 0.5 ml proteinase K solution (2 mg ml^−1^ proteinase K, 10 mM Tris-HCl pH 7.5, 100 mM EDTA, 0.5% SDS, 20 mM NaCl) overnight at 50 °C, washed (2 × 1 h) in TE50 solution (10 mM Tris-HCl pH 7.5, 50 mM EDTA, 0.5% SDS, 100 mM NaCl), (2 × 1 h) in TE buffer solution (10 mM Tris-HCl pH 7.5, 1 mM EDTA, 100 mM NaCl) and then incubated in 1 ml of MES (2-(N-morpholino)ethanesulfonic acid) solution (35 mM MES hydrate, 150 mM MES sodium salt, 100 mM NaCl) for 20 min at 68 °C. The tubes were cooled at 42 °C for 10 min before addition of 3 µl of β-agarase (NEB) dissolved in 100 µl MES solution and incubation overnight at 42 °C. The samples were then carefully poured into combing reservoirs containing 1.2 ml of MES solution supplemented with 2 mM Zn(O_2_CCH_3_)_2_ and either S1 nuclease (40 U ml^−1^) or S1 nuclease dilution buffer (Thermo Fisher) and incubated for 30 min at room temperature. Next, genomic DNA was combed onto silanized coverslips (Genomic Vision) using a combing machine (Genomic Vision) and coverslips baked for 2 h at 60 °C. DNA was denatured in fresh 0.5 M NaOH solution containing 1 M NaCl for 8 min at room temperature and coverslips then washed (3 × 3 min) in PBS. Coverslips were then incubated in blocking solution (1% BSA with 0.1% Tween20 in PBS) for 30 min at room temperature and subsequently stained with antibodies at 37 °C in a humid chamber. Coverslips were first incubated with primary rat monoclonal anti-BrdU (Abcam, ab6326; 1:50) and mouse monoclonal anti-BrdU (Becton Dickinson, 347580; 1:25) for 1 h, followed by incubation with secondary goat anti-mouse Alexa Fluor 488 (Thermo Fisher, A11001; 1:25) and goat anti-rat Alexa Fluor 568 (Thermo Fisher, A11077; 1:25) for 45 min. Coverslips were then incubated with mouse monoclonal anti-ssDNA antibody (Millipore, MAB3034; 1:25) for 2 h to stain all genomic DNA and subsequently with donkey anti-mouse Alexa Fluor 647 (Thermo Fisher, A31571; 1:25) for 45 min. Coverslips were washed (3 × 3 min) in PBS with Tween after each antibody incubation. Finally, coverslips were dried and mounted onto microscope slides in fluoroshield (Merck), and high-resolution images acquired with an Apotome widefield microscope (Zeiss) using either ×40 or ×63 oil objectives. ImageJ64 software (NIH, https://imagej.nih.gov/ij/) was used to measure lengths of labeled replication tracks. The speed of replication fork progression was calculated assuming a constant stretching factor of 2 kb µm^−1^.

### Flow cytometry

DT40 cells (roughly 2 × 10^6^ per sample) were collected, washed and resuspended in 100 µl of ice-cold PBS. Next, 900 µl of 70% ethanol was added to the cell suspension dropwise while gently vortexing and samples were incubated in fixing solution overnight or longer at 4 °C. Before analysis, cells were washed in PBS and stained in the dark with 500 µl of PBS solution (2 mM MgCl_2_, 50 µg ml^−1^ propidium iodide, 50 µg ml^−1^ RNase A) for 20 min at 37 °C. Cells were counted using BD Accuri C6 Plus Flow Cytometer. The data were analyzed and visualized using FlowJo software (FlowJo LLC, https://www.flowjo.com/).

### Electron microscopy

For electron microscopy, roughly 0.5 × 10^8^ DT40 cells were treated with DMSO or 10 µM PARP inhibitor (KU0058948 hydrochloride, Axon Medchem, Axon 2001) for 1 h at +37 °C. Cells were then placed and processed on ice for all subsequent steps unless otherwise indicated. Genomic DNA was cross-linked in vivo by triple incubation with 10 µg ml^−1^ 4,5′,8-trimethylpsoralen (Merck, T6137) for 5 min followed by irradiation with 365-nm wavelength ultraviolet light for 7 min on a precooled metal^57,58^. Cells were then lysed in buffer containing 1.28 M sucrose, 40 mM Tris-HCl pH 7.5, 20 mM MgCl_2_, 4% Triton X-100 and 10 µM of both PARP inhibitor and PARG inhibitor (PDD00017273, Merck, SML1781) to prevent PARP/PARG activity postlysis. Nuclei were then pelleted (1,300*g*, 15 min, 4 °C) and digested in buffer containing 800 mM GdmCl, 30 mM Tris-HCl pH 8.0, 30 mM EDTA, 5% Tween20, 0.5% Triton X-100 and proteinase K (Thermo Fisher Scientific, EO0492). Genomic DNA was extracted with chloroform/isoamylalcohol, precipitated with isopropanol and resuspended in TE buffer (10 mM Tris-HCl pH 8.0, 1 mM EDTA). DNA was then digested with *Pvu*II HF (New England Biolabs, R3151S; 33 U per 10 µg DNA) supplemented with 33.3 µg of RNase A (Thermo Fisher Scientific, EN0531) and 0.013U ShortCut RNase III (New England Biolabs, M0245S) in the CutSmart restriction buffer (New England Biolabs, B7204S) for 3 h at +37 °C. The digested DNA was then concentrated and recovered using Microcon DNA Fast Flow Centrifugal Filters (Merck, MRCF0R100). The DNA was then spread on a water surface using the benzyldimethylalkylammonium chloride method and transferred on the carbon-coated 400 mesh copper grids (Plano, G2400C). Next, DNA was platinum coated using a Leica EM ACE900 sample preparation system. The grids were examined using Jeol JEM-1400 Flash transmission electron microscope operated at ≤120 kV (0.2 nm resolution) and the images were acquired with Jeol Flash 2,000 × 2,000 pixels CMOS camera. For the automatic acquisition, we used Limitless Panorama or Serial EM capturing modes. Replication forks were analyzed using ImageJ64 (NIH). For analysis, only spatially separated unambiguous fork structures with clear ultrastructural characteristics (a fork junction with parental and two daughter helices) were scored. For each experimental condition, 20 replication forks were analyzed in each of two independent biological replicates.

### Statistics and reproducibility

All statistics used GraphPad Prism (v.9.1) unless stated otherwise, using the tests indicated in the figure legends. Where possible, hierarchical/nested analyses were conducted with matching within experimental repeats. *n* reflects the number of independent experimental/biological repeats, whereas measurements of individual cells (comet tail moments and DNA fiber lengths) within each experiment were treated as technical repeats.

### Reporting Summary

Further information on research design is available in the Nature Research Reporting Summary linked to this article.

## Online content

Any methods, additional references, Nature Research reporting summaries, source data, extended data, supplementary information, acknowledgements, peer review information; details of author contributions and competing interests; and statements of data and code availability are available at 10.1038/s41594-022-00747-1.

## Supplementary information

Reporting Summary

Peer Review Information

## Data Availability

All raw data are present online as Source data files provided with this paper.

## Source data

Source Data Fig. 1 Uncropped blots for Fig. 1b.
Source Data Fig. 1 Quantitative data for Fig. 1.
Source Data Fig. 2 Quantitative data for Fig. 2 (these are also the data for Extended Data Fig. 2a,b,d: Fig. 2 shows the combined data and Extended Data Fig. 2a,b,d shows the data from the individual experiments).
Source Data Fig. 3 Quantitative data for Fig. 3.
Source Data Fig. 4 Quantitative data for Fig. 4 (these are also the data for Extended Data Fig. 3a–c: Fig. 4 shows the combined data and Extended Data Fig. 3a–c shows the data from the individual experiments).
Source Data Fig. 5 Quantitative data for Fig. 5.
Source Data Fig. 6 Quantitative data for Fig. 6 (these are also the data for Extended Data Fig. 5b,c: Fig. 6 shows the combined data and Extended Data Fig. 5b,c shows the data from the individual experiments).
Source Data Extended Data Fig. 1 Quantitative data for Extended Data Fig. 1.
Source Data Extended Data Fig. 2 Quantitative data for Extended Data Fig. 2c,e.
Source Data Extended Data Fig. 3 Quantitative data for Extended Data Fig. 3d.
Source Data Extended Data Fig. 4 Uncropped blots for Extended Data Fig. 4a.
Source Data Extended Data Fig. 5 Quantitative data for Extended Data Fig. 5a.
Source Data Extended Data Fig. 6 Quantitative data for Extended Data Fig. 6a.
Source Data Extended Data Fig. 6 Uncropped blots for Extended Data Fig. 6b.

## Extended data

are available for this paper at 10.1038/s41594-022-00747-1.
