# Supplementary material for: PARP inhibition impedes the maturation of nascent DNA strands during DNA replication
Source: Nat Struct Mol Biol. 2022 Mar 24;29(4):329–38. doi: 10.1038/s41594-022-00747-1 (PMC9010290; doi:10.1038/s41594-022-00747-1)
Supplement: Supplementary file 1 — Reporting Summary [file 41594_2022_747_MOESM1_ESM.pdf]

nature portfolio

Corresponding author(s): keith caldecott

Last updated by author(s): 27th Jan 2022

Reporting Summary

Nature Portfolio wishes to improve the reproducibility of the work that we publish. This form provides structure for consistency and transparency in reporting. For further information on Nature Portfolio policies, see our Editorial Policies and the Editorial Policy Checklist.

Statistics

For all statistical analyses, confirm that the following items are present in the figure legend, table legend, main text, or Methods section.

n/a	Confirmed	
☐	☒	The exact sample size (*n*) for each experimental group/condition, given as a discrete number and unit of measurement
☐	☒	A statement on whether measurements were taken from distinct samples or whether the same sample was measured repeatedly
☐	☒	The statistical test(s) used AND whether they are one- or two-sided<br>*Only common tests should be described solely by name; describe more complex techniques in the Methods section.*
☐	☒	A description of all covariates tested
☐	☒	A description of any assumptions or corrections, such as tests of normality and adjustment for multiple comparisons
☐	☒	A full description of the statistical parameters including central tendency (e.g. means) or other basic estimates (e.g. regression coefficient) AND variation (e.g. standard deviation) or associated estimates of uncertainty (e.g. confidence intervals)
☐	☒	For null hypothesis testing, the test statistic (e.g. *F*, *t*, *r*) with confidence intervals, effect sizes, degrees of freedom and *P* value noted<br>*Give P values as exact values whenever suitable.*
☒	☐	For Bayesian analysis, information on the choice of priors and Markov chain Monte Carlo settings
☐	☒	For hierarchical and complex designs, identification of the appropriate level for tests and full reporting of outcomes
☒	☐	Estimates of effect sizes (e.g. Cohen's *d*, Pearson's *r*), indicating how they were calculated

Our web collection on statistics for biologists contains articles on many of the points above.

Software and code

Policy information about availability of computer code

Data collection	Comet Assay IV (software version 4.1) was employed for acquisition of single cell comet tail moments, and ScanR (software version 3.2.0) for high content microscope image acquisition
Data analysis	Comet Assay IV was employed for data analysis of single cell comet tail moments, and ScanR & Image J64 (software version 2.1.0/1.53c) for analysis of microscope images

For manuscripts utilizing custom algorithms or software that are central to the research but not yet described in published literature, software must be made available to editors and reviewers. We strongly encourage code deposition in a community repository (e.g. GitHub). See the Nature Portfolio guidelines for submitting code & software for further information.

Data

Policy information about availability of data

All manuscripts must include a data availability statement. This statement should provide the following information, where applicable:

- Accession codes, unique identifiers, or web links for publicly available datasets
- A description of any restrictions on data availability
- For clinical datasets or third party data, please ensure that the statement adheres to our policy

Data availability. All raw data are present online as Source data files.

Field-specific reporting

Please select the one below that is the best fit for your research. If you are not sure, read the appropriate sections before making your selection.

☒ Life sciences ☐ Behavioural & social sciences ☐ Ecological, evolutionary & environmental sciences

For a reference copy of the document with all sections, see nature.com/documents/nr-reporting-summary-flat.pdf

Life sciences study design

All studies must disclose on these points even when the disclosure is negative.

Sample size	For comet assays/fibre assays, we score at least 50-100 single cells (technical replicates) per experiment, and typically conduct 3 or more biological repeats (N=>3). This number of technical replicates satisfies the central limit theorem for assumptions of normality and the biological repeats (n=>3) enable robust statistical analysis of experimental reproducibility.
Data exclusions	no data points are excluded
Replication	Experiments typically involve 3 (or more) biological repeats. All such replicates are included in our data sets and statistical analysis.
Randomization	Randomisation and covariates were not applicable in our study, because we are using genetically defined cell lines cultured together in parallel under identical conditions. Moreover, we employed a hierarhical design, in which different genetically defined cell lines are blocked together within each independent experimental repeat, thereby controlling for experimental variation.
Blinding	Experimenter blinding was not employed for these experiments, because cells are scored randomly using automated image acquisition software such as ScanR high content microscopy and thus independently of user bias.

Reporting for specific materials, systems and methods

We require information from authors about some types of materials, experimental systems and methods used in many studies. Here, indicate whether each material, system or method listed is relevant to your study. If you are not sure if a list item applies to your research, read the appropriate section before selecting a response.

Materials & experimental systems

n/a	Involved in the study	
☐	☒	Antibodies
☐	☒	Eukaryotic cell lines
☒	☐	Palaeontology and archaeology
☒	☐	Animals and other organisms
☒	☐	Human research participants
☒	☐	Clinical data
☒	☐	Dual use research of concern

Methods

n/a	Involved in the study	
☒	☐	ChIP-seq
☒	☐	Flow cytometry
☒	☐	MRI-based neuroimaging

Antibodies

Antibodies used	Primary antibodies: Anti-poly-ADP-ribose (PAR) binding reagent (Millipore, MABE1031), rabbit anti-mono/poly-ADP ribose Mab (PAR/MAR; Cell Signaling, CST 83732), mouse anti-PCNA Mab (Santa Cruz, sc-56), rabbit anti-PARP1 Mab (Cell Signaling, 9532), rat anti-α-tubulin polyclonal (Abcam, ab6160), rabbit anti-H3 polyclonal (Abcam, ab1791), rabbit anti-FEN1 polyclonal (LifeSpan Biosciences, LS-C80825), mouse anti-FEN1 Mab (Invitrogen, MA1-23228), mouse anti-biotin Mab (Merck, BN-34), mouse anti-PARP1 Mab (Santa Cruz, sc-8007), rat recombinant anti-PCNA (Abcam, ab252848), rat anti-BrdU Mab (Abcam, ab6326), mouse anti-BrdU Mab (Becton Dickinson, 347580), mouse anti-ssDNA Mab (Millipore, MAB3034), mouse anti-RPA2 Mab (Abcam, ab2175), rabbit anti-RPA2 pS33 polyclonal (NB100-544, Novus Biologicals), rabbit anti-RPA2 pS4/8 polyclonal (Millipore, PLA0071), mouse anti-importin [illegible] Mab (Santa Cruz, sc-137016).<br>Secondary antibodies: HRP-conjugated goat anti-rabbit (Bio-Rad, 170-6515), HRP-conjugated goat anti-mouse (Bio-Rad, 170-6516, HRP-conjugated rabbit anti-rat (Abcam, ab6734), donkey anti-rabbit Alexa Fluor 488 (Thermo Fisher, A21206), donkey anti-mouse Alexa Fluor 568 (Thermo Fisher, A10037), donkey anti-mouse Alexa Fluor 647 (Thermo Fisher, A31571), goat anti-mouse Alexa Fluor 488 (Thermo Fisher, A11001), goat anti-mouse Alexa Fluor 488 (Thermo Fisher, A32723), donkey anti-goat Alexa Fluor 488 (Thermo Fisher, A11055), goat anti-rat Alexa Fluor 568 (Thermo Fisher, A11077), donkey anti-rat Alexa Fluor 488 (Thermo Fisher, A21208).
Validation	Antibodies were validated either by the manufacturer (as indicated on their website) and/or by previous publications and/or by ourselves in this study by the inclusion of relevant gene-edited cell lines lacking the antigen in question (e.g. FEN1, PARP1/PAR/MAR)

Eukaryotic cell lines

Policy information about cell lines

Cell line source(s)	RPE-1 & U2OS (both from ATCC); FEN1-/- DT40 cells obtained from Matsuzaki et al (Nuclic Acids Research 30: 3273-3277, 2002).
Authentication	Verified routinely in our Centre by genetic fingerprinting
Mycoplasma contamination	All cell lines are mycoplasma negative and verified as such in our Centre routine testing
Commonly misidentified lines (See ICLAC register)	none
