# Supplementary figures and images for: PARP inhibition impedes the maturation of nascent DNA strands during DNA replication
Source: Nat Struct Mol Biol. 2022 Mar 24;29(4):329–38. doi: 10.1038/s41594-022-00747-1 (PMC9010290; doi:10.1038/s41594-022-00747-1)

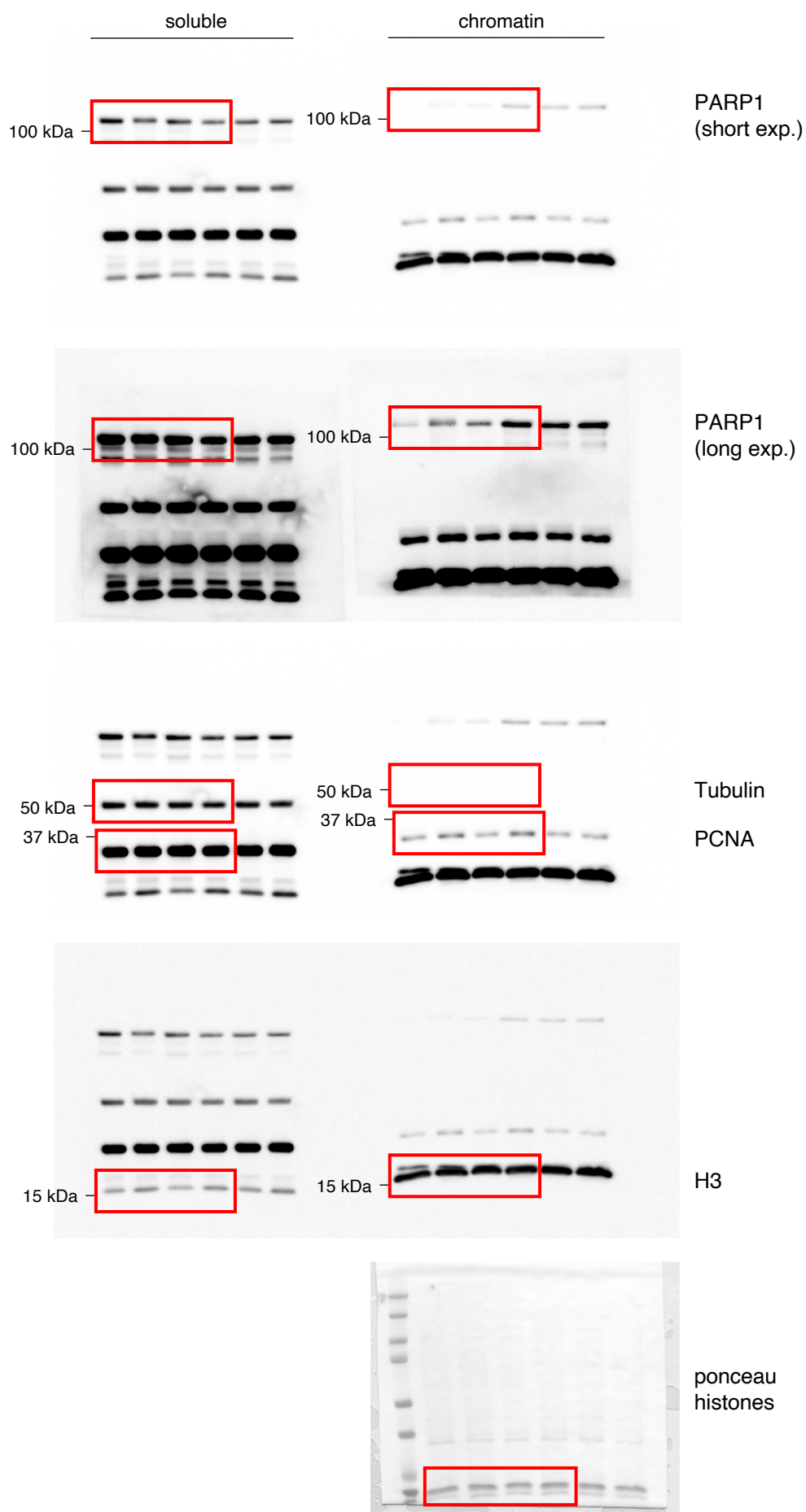

**Figure 1B**

Supplement: Source Data Fig. 1 — Uncropped blots for Fig. 1b. [file 41594_2022_747_MOESM3_ESM.pdf]

Extended figure 4a

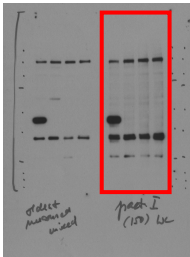

FEN1  
(Invitrogen)

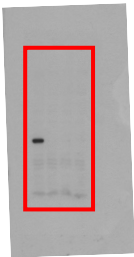

FEN1  
(LifeSpan Biosciences)

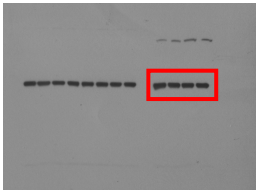

β-tubulin

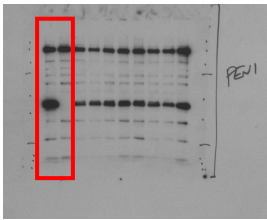

FEN1  
(Invitrogen)

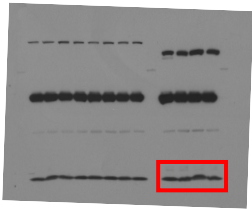

H3

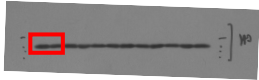

H3

Supplement: Source Data Extended Data Fig. 4 — Uncropped blots for Extended Data Fig. 4a. [file 41594_2022_747_MOESM13_ESM.pdf]

Extended figure 6b

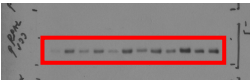

pRPA2 S33

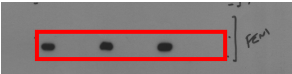

FEN1 (Invitrogen)

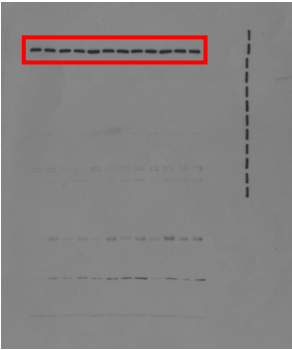

importin  $\beta$

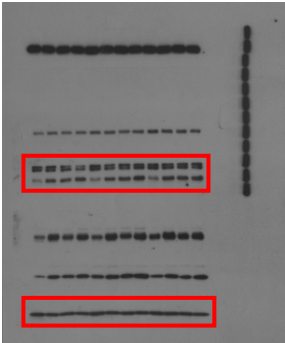

pRPA2 S4/8

H3

Supplement: Source Data Extended Data Fig. 6 — Uncropped blots for Extended Data Fig. 6b. [file 41594_2022_747_MOESM16_ESM.pdf]
