# Supplementary material for: PARP inhibition impedes the maturation of nascent DNA strands during DNA replication
Source: Nat Struct Mol Biol. 2022 Mar 24;29(4):329–38. doi: 10.1038/s41594-022-00747-1 (PMC9010290; doi:10.1038/s41594-022-00747-1)
Supplement: Supplementary file 2 — Peer Review Information [file 41594_2022_747_MOESM2_ESM.pdf]

natureresearch

Peer Review Information

Journal: Structural and Molecular Biology
Manuscript Title: PARP Inhibition Impedes the Maturation of Nascent DNA Strands During DNA Replication
Corresponding author name(s): Professor Keith Caldecott, Dr Hana Hanzlikova

Editorial Notes:

**Redactions – unpublished data**	Parts of this Peer Review File have been redacted as indicated to maintain the confidentiality of unpublished data.
**Redactions – confidential patient information**	Parts of this Peer Review File have been redacted as indicated to maintain patient confidentiality.
**Redactions – published data**	Parts of this Peer Review File have been redacted as indicated to remove third-party material.
**Redactions – reviewer opt-out**	Parts of this Peer Review File have been redacted as indicated as we could not obtain permission to publish the reports of reviewer no. XX.
**Reviewer comments in marked-up manuscript**	In their review of the [first/second/third/...] version of this manuscript, reviewer no. XX added their comments to the manuscript file. These comments, excluding minor textual revisions, have been copied into this Peer Review File.

Reviewer Comments & Decisions:

Decision Letter, initial version:

16th Aug 2021

Dear Keith,

Thank you again for submitting your manuscript "PARP Inhibition Impedes the Maturation of Nascent DNA Strands During DNA Replication". I apologize for the delay, due largely to the summer holidays, while we awaited comments (below) from the 2 reviewers who evaluated your paper. In light of their reports, we remain interested in your study and would like to see your response to the comments of the

referees, in the form of a revised manuscript.

You will see that while the reviewers (both experts in DNA replication and genome instability) are positive about the potential interest of the findings, they each make suggestions to improve the work. Reviewer #1 raises several clarification points that should be addressed by text revisions. Reviewer #2 requests additional experimentation to demonstrate that the observed effects are due solely to lagging strand maturation, and also highlights some supporting data that need to included as well as aspects of the work that must be discussed in the context of the literature. Editorially, we agree that these suggestions would strengthen the study, and ask that they be included in a revised manuscript.

Please be sure to address/respond to all concerns of the referees in full in a point-by-point response and highlight all changes in the revised manuscript text file. If you have comments that are intended for editors only, please include those in a separate cover letter.

We are committed to providing a fair and constructive peer-review process. Do not hesitate to contact us if there are specific requests from the reviewers that you believe are technically impossible or unlikely to yield a meaningful outcome.

We expect to see your revised manuscript within 6 weeks. If you cannot send it within this time, please contact us to discuss an extension; we would still consider your revision, provided that no similar work has been accepted for publication at NSMB or published elsewhere.

As you already know, we put great emphasis on ensuring that the methods and statistics reported in our papers are correct and accurate. As such, if there are any changes that should be reported, please submit an updated version of the Reporting Summary along with your revision.

Please follow the links below to download these files:

Reporting Summary:
https://www.nature.com/documents/nr-reporting-summary.pdf

Please note that the form is a dynamic 'smart pdf' and must therefore be downloaded and completed in Adobe Reader.

When submitting the revised version of your manuscript, please pay close attention to our href="https://www.nature.com/nature-research/editorial-policies/image-integrity">Digital Image Integrity Guidelines. and to the following points below:

-- that unprocessed scans are clearly labelled and match the gels and western blots presented in figures.
-- that control panels for gels and western blots are appropriately described as loading on sample processing controls
-- all images in the paper are checked for duplication of panels and for splicing of gel lanes.

Finally, please ensure that you retain unprocessed data and metadata files after publication, ideally archiving data in perpetuity, as these may be requested during the peer review and production process or after publication if any issues arise.

Please note that all key data shown in the main figures as cropped gels or blots should be presented in uncropped form, with molecular weight markers. These data can be aggregated into a single supplementary figure item. While these data can be displayed in a relatively informal style, they must refer back to the relevant figures. These data should be submitted with the final revision, as source data, prior to acceptance, but you may want to start putting it together at this point.

SOURCE DATA: we urge authors to provide, in tabular form, the data underlying the graphical representations used in figures. This is to further increase transparency in data reporting, as detailed in this editorial (http://www.nature.com/nsmb/journal/v22/n10/full/nsmb.3110.html). Spreadsheets can be submitted in excel format. Only one (1) file per figure is permitted; thus, for multi-paneled figures, the source data for each panel should be clearly labeled in the Excel file; alternately the data can be provided as multiple, clearly labeled sheets in an Excel file. When submitting files, the title field should indicate which figure the source data pertains to. We encourage our authors to provide source data at the revision stage, so that they are part of the peer-review process.

Data availability: this journal strongly supports public availability of data. All data used in accepted papers should be available via a public data repository, or alternatively, as Supplementary Information. If data can only be shared on request, please explain why in your Data Availability Statement, and also in the correspondence with your editor. Please note that for some data types, deposition in a public repository is mandatory - more information on our data deposition policies and available repositories can be found below:
https://www.nature.com/nature-research/editorial-policies/reporting-standards#availability-of-data

We require deposition of coordinates (and, in the case of crystal structures, structure factors) into the Protein Data Bank with the designation of immediate release upon publication (HPUB). Electron microscopy-derived density maps and coordinate data must be deposited in EMDB and released upon publication. Deposition and immediate release of NMR chemical shift assignments are highly encouraged. Deposition of deep sequencing and microarray data is mandatory, and the datasets must be released prior to or upon publication. To avoid delays in publication, dataset accession numbers must be supplied with the final accepted manuscript and appropriate release dates must be indicated at the

galley proof stage.

Please use the link below to submit your revised manuscript and related files:

[REDACTED]

Note: This URL links to your confidential home page and associated information about manuscripts you may have submitted, or that you are reviewing for us. If you wish to forward this email to co-authors, please delete the link to your homepage.

We look forward to seeing the revised manuscript and thank you for the opportunity to review your work.

With kind regards,

Beth

Beth Moorefield, Ph.D.
Senior Editor
Nature Structural & Molecular Biology

Reviewers' Comments:

Reviewer #1:
Remarks to the Author:
Poly ADP-ribosylation (PAR) is a post-translational modification that regulates numerous biological processes. Caldecott's group has recently shown that unligated Okazaki fragments are a major source of Parylation during DNA replication. By using a variety of complementary techniques such as BrdU comet, alkalyne gel electrophoresis and DNA fiber assays in chicken and human cells, they show in the current manuscript that PARP inhibitors interfere with Okazaki fragment maturation in S-phase, thereby implicating unligated Okazaki fragments as major sources of PARP1 trapping cytotoxicity. The findings presented here are of considerable interest and provide a convincing explanation for PARPi cytotoxicity. I would recommend publication in NSMB after a few clarifications.

- The evidence that PARPi affecting OF maturation in WT cells mainly comes from BrdU comet assays. Other experiments (Parylation quantification in S-phase, comet in S-phase, alkalyne gel electrophoresis, DNA fibers treated with S1 nuclease) do not show clear evidence that PARPi influence nascent DNA in WT cells. Can the authors comment on this?

- They discuss that in WT cells, maturation is slowed down at 10-100 kb distances, whereas in FEN1-/-, Okazaki fragment maturation is impacted much closer to the replication fork. It would be useful for the authors to explain why this might be the case- and particularly helpful might be a model figure in the paper. It is not clear to me why PARP activity is normally
greatest at considerable distances behind DNA replication.
- The fork speed is similar in WT and FEN1 KO cells, yet the doubling time in the mutant is considerably slower. Can the authors provide an explain this?
- Authors performed PLA experiments in Fig 5 to detect proximity of PAR and EdU. When incubating cells with EdU for 20-30min and performing Click IT reaction with Azide coupled with fluorophores (such as Alexa 488/555), EdU normally shows a widespread signal in the nucleus in majority of S-phase cells- only few show a punctuate pattern. Is it possible that this widespread EdU signal increases the background in PLA assays? Would a shorter incubation time help?
- Why do the authors add 10uM of PARPi during the pre-extraction even if cells are not treated with PARPi during the PLA experiments?

Minor:

Change text - Fig2C to 2D in lanes 145 and 147.

Reviewer #2:
Remarks to the Author:
The article by Vaitsiankova et al. describes a set of analyses probing the role of PARP in DNA replication – more specifically the maturation of Okazaki fragments on the nascent lagging strand. They propose that PARP activity is required for the timely maturation of nascent DNA, and suggest that this function of PARP might be a major determinant of how PARP inhibitors generate cell toxicity. Given the importance of PARP inhibitors in the treatment of certain human cancers, the data presented could be important not only for our understanding of a fundamental cellular process, but also for our ability to selectively target cancer cells. Overall, the article is interesting and provocative, although I felt that the evidence supporting some the conclusions needs to be strengthened.

Specific comments:

Major issues

1) I could not understand why PARP activity would be highest tens of kilobases behind replication forks. Maturation of lagging strand synthesis is generally quite rapid and occurs in concert with the moving

fork. Having ssDNA gaps at a great distance from the fork implies perhaps a role in gap filling by TLS or HR rather than strictly Okazaki fragment processing.
2) What is the evidence that the events studied are even located on the nascent lagging strand?
3) I don't feel the evidence presented that the effect is caused specifically by changes in Okazaki fragment maturation is strong enough. For example, my understanding is that an uncoupling of leading and lagging strand synthesis, as has been seen in other studies, could give rise to the effects seen here. Can the authors provide more evidence to substantiate their conclusions?
4) Most of the study utilizes a FEN1-/- cell line, with the implication that this affects lagging strand processing. However, while that is likely to be the case, FEN1 plays roles in DNA repair pathways as well. Using an alternative system to link the effects solely to lagging strand maturation would be helpful.

Other comments:

1) I can't understand why S-phase ADP-ribosylation is not increased in FEN1-/- DT40 cells without the need for a PARG inhibitor – to say that it is increased but 'fell short of statistical significance' really means it wasn't increased!
2) In Figure 1B, the Ponceau staining should be shown, particularly as there is a concern that the PCNA levels seem to mirror those of PARP1, implying perhaps a loading issue.
3) The authors should utilize RPA staining to substantiate analysis of ssDNA gap formation being specific, this could be combined with EdU analysis to define newly synthesized DNA. On a similar note, blotting for RPA S4/8 phosphorylation should define whether general replication is perturbed with the formation of ssDNA.
4) The recent paper from the Bartek group (ref#31) indicated that PARP inhibition affects fork speed quite noticeably. That was a major paper in the field and highly relevant to the current manuscript because, if correct, it would alter the interpretation of the current data. The Bartek work is 'dismissed' in a single sentence, which is a bit unsatisfactory. The authors propose that the time period of PARP inhibition might explain the difference. I suggest that they test this proposal directly and, if correct, then their claims would be far stronger.
5) In an ideal world, although I realize that these things are laborious and complex to conduct, the authors would strain their fibers for ssDNA and/or analyze nascent ssDNA gap formation using electron microscopy.
6) In Figure 5B, there should be a statistical comparison between the WT and FEN1-/- samples at each time point. Are they really different?

Author Rebuttal to Initial comments

Reviewer #1:
Remarks to the Author: Poly ADP-ribosylation (PAR) is a post-translational modification that regulates numerous biological processes. Caldecott's group has recently shown that unligated Okazaki fragments are a major source of Parylation during DNA replication. By using a variety of complementary techniques such as BrdU comet, alkalyne gel

electrophoresis and DNA fiber assays in chicken and human cells, they show in the current manuscript that PARP inhibitors interfere with Okazaki fragment maturation in S-phase, thereby implicating unligated Okazaki fragments as major sources of PARP1 trapping cytotoxicity. The findings presented here are of considerable interest and provide a convincing explanation for PARPi cytotoxicity. I would recommend publication in NSMB after a few clarifications.

- The evidence that PARPi affecting OF maturation in WT cells mainly comes from BrdU comet assays. Other experiments (Parylation quantification in S-phase, comet in S-phase, alkalyne gel electrophoresis, DNA fibers treated with S1 nuclease) do not show clear evidence that PARPi influence nascent DNA in WT cells. Can the authors comment on this?

This is a good point. We believe the ability of comet assays to detect the impact of PARPi on the integrity in WT cells reflects the sensitivity of this assay to very low numbers of unrepaired SSBs. For example, because our nascent strand comet assays are sensitive to fragment sizes >500kb, this assay can in principle detect a single unrepaired OF present every 1000 Kb fragment. In contrast, such a frequency of unrepaired SSBs cannot be detected easily by assays that measure fragments of <50kb (such as fibre combing or slab electrophoresis) in which only ~5% of the fragments would be affected. However, if both PARP1 and FEN1-dependent repair is perturbed, far more SSBs persist during the 30-40 min pulse/chase period and so are detected in the smaller (<50kb) fragments. Note however, that we have now confirmed that gaps are created/persist in the presence of PARPi close to the fork (within 2kb) using electron microscopy, which can capture a 'snapshot' of gaps present close to the fork before they are repaired (Figure 4E).

- They discuss that in WT cells, maturation is slowed down at 10-100 kb distances, whereas in FEN1-/-, Okazaki fragment maturation is impacted much closer to the replication fork. It would be useful for the authors to explain why this might be the case- and particularly helpful might be a model figure in the paper. It is not clear to me why PARP activity is normally greatest at considerable distances behind DNA replication.

We apologise for not explaining this clearly. Our model is that PARP1 detects OF's that have escaped canonical (FEN1-dependent) processing, and which have thus been left behind the replisome (hence the higher PARP1 activity behind the fork). We propose that PARP1 detects unligated OF's over a broad range of distances behind the fork, from within several kilobases to hundreds of kilobases. However, the impact of PARP1 is more evident in FEN1-/- cells closer to the fork because there are far more OF's escaping canonical processing. This is now clarified in the discussion section on page 9 (highlighted in yellow). In addition, we have added a diagrammatic model in Fig.6D, as requested.

- The fork speed is similar in WT and FEN1 KO cells, yet the doubling time in the mutant is considerably slower. Can the authors provide an explain this?

Indeed, it has been reported previously that FEN1-/- cells exhibit increased apoptosis (Matsuzaki et al NAR, 2002), which affects the doubling time of this strain. We have also observed this in our experiments, as indicated by an increased sub-G1 population. These data are shown in Extended Data Fig.1A.

- Authors performed PLA experiments in Fig 5 to detect proximity of PAR and EdU. When incubating cells with EdU for 20-30min and performing Click IT reaction with Azide coupled with fluorophores (such as Alexa 488/555), EdU normally shows a widespread signal in the nucleus in majority of S-phase cells- only few show a punctuate pattern. Is it possible that this widespread EdU signal increases the background in PLA assays? Would a shorter incubation time help?

This is a good point. The focal PLA signal is unlikely to reflect the level of EdU because it decreases during the chase period, whereas the level of EdU remains the same. Rather, the sites of focal PLA signal likely reflect the very short pulse labelling period with EdU (10 min), and/or possibly high focal levels of PAR (e.g. perhaps at sites with high local levels of unligated OF's).

- Why do the authors add 10uM of PARPi during the pre-extraction even if cells are not treated with PARPi during the PLA experiments?

This is to prevent the ‘spurious’ activation of PARP upon cell lysis, which would otherwise result in increased ‘background’ PARylation.

Minor:
Change text - Fig2C to 2D in lanes 145 and 147.

Thank you. We have changed the text to “Fig.2C, right…” instead (there is no Fig.2D in this figure).

Reviewer #2:
Remarks to the Author: The article by Vaitsiankova et al. describes a set of analyses probing the role of PARP in DNA replication – more specifically the maturation of Okazaki fragments on the nascent lagging strand. They propose that PARP activity is required for the timely maturation of nascent DNA, and suggest that this function of PARP might be a major determinant of how PARP inhibitors generate cell toxicity. Given the importance of PARP inhibitors in the treatment of certain human cancers, the data presented could be important not only for our understanding of a fundamental cellular process, but also for our ability to selectively target cancer cells. Overall, the article is interesting and provocative, although I felt that the evidence supporting some the conclusions needs to be strengthened.

Specific comments:
Major issues

1) I could not understand why PARP activity would be highest tens of kilobases behind replication forks. Maturation of lagging strand synthesis is generally quite rapid and occurs in concert with the moving fork. Having ssDNA gaps at a great distance from the fork implies perhaps a role in gap filling by TLS or HR rather than strictly Okazaki fragment processing.

We apologise for not explaining this clearly. We propose that PARP1 detects unligated OF’s over a broad range of distances behind the fork, from within several kilobases to hundreds of kilobases. The impact of PARP1 close to the fork becomes more evident in FEN1-/- cells, as indicated by our alkaline gel electrophoresis and fibre experiments, consistent with more OFs escaping canonical processing and so increasing the likelihood of PARP1 activation closer to the fork. This is now clarified in the discussion section on page 8 (highlighted in yellow), and we have added a model in Fig.6D.

In terms of the possible relationship of S phase PARP activity with TLS or HR, we have now addressed this directly, by examining the impact of inhibiting these pathways on levels of S phase PAR. However, we find that TLS inhibitor (the REV1 inhibitor JH-RE-06) and BRCA1 deletion have little effect on level of S phase PAR in human cells. Whilst we cannot rule out an involvement of these pathways in PARP activation in S phase, our data do not support such a model. These data are now added in Extended Data Figure 5A.

2) What is the evidence that the events studied are even located on the nascent lagging strand?
3) I don’t feel the evidence presented that the effect is caused specifically by changes in Okazaki fragment maturation is strong enough. For example, my understanding is that an uncoupling of leading and lagging strand synthesis, as has been seen in other studies, could give rise to the effects seen here. Can the authors provide more evidence to substantiate their conclusions?

4) Most of the study utilizes a FEN1-/- cell line, with the implication that this affects lagging strand processing. However, while that is likely to be the case, FEN1 plays roles in DNA repair pathways as well. Using an alternative system to link the effects solely to lagging strand maturation would be helpful.

We have responded to comments 2-4 together, since they relate to the same issue. These are all valid and good points. Indeed, it is very difficult to prove that PARP1 is primarily activated during S phase on lagging strands, because of the essential nature of this process and because, to our knowledge, there isn't any protein that can be manipulated that plays a role solely in lagging strand maturation. The evidence that the majority of S phase PARP activity is a result of unligated Okazaki fragments was presented in our recent Mol Cell paper (Hanzlikova et al Mol Cell 2018). Of particular relevance to the current manuscript was our demonstration that none of the S phase DNA repair pathways that might generate incision products that could activate PARP1 affected the level of S phase PARP activity. We showed this by demonstrating that levels of S phase PARP activity were similar in wild type cells and cells lacking the proteins that mediated different pathways of S phase DNA excision repair.

To present more evidence in the current paper we have now applied the same approach to show that the greatly increased S phase PARP activity triggered by FEN1 inhibitor is not due to SSBs arising during either ribonucleotide excision repair or base excision repair, which are the two known DNA excision repair pathways operative in S phase that employ FEN1. To do this we have employed cells lacking either RNASEH2 (which creates incisions during ribonucleotide excision repair) or APE1 (which creates incisions during base excision repair). In the latter case we also employed XRCC1 deletion to increase the half-life of SSBs and thereby the sensitivity of the assay. These data are now presented in Extended Data Fig.1C. However, whilst our data strongly implicate OFs as the major source of S phase PARP activity in wild type and FEN1-/- cells, we do not rule out a contribution from other DNA structures. We have now discussed this issue in the text (Page 8/9, highlighted in yellow).

Other comments:

1) I can't understand why S-phase ADP-ribosylation is not increased in FEN1-/- DT40 cells without the need for a PARG inhibitor – to say that it is increased but 'fell short of statistical significance' really means it wasn't increased!

Yes, this was a little unsatisfying we agree, especially since the level of S phase PAR was significantly higher in three independent human U2OS FEN1-/- KO clones as well as our new RPE-1 FEN1-/- KO clone even in the absence of PARGi (see new Fig. 5A). The mean PAR intensity in *FEN1*$^{-/-}$ DT40 cells is 3-fold higher than in wild type DT40, but the experimental variation across experiments prevented the p-value from reaching the 0.05 threshold. We have therefore now conducted additional experiments to increase statistical power, and the 3-fold difference is statistically significant (see new Fig.1A).

2) In Figure 1B, the Ponceau staining should be shown, particularly as there is a concern that the PCNA levels seem to mirror those of PARP1, implying perhaps a loading issue.

We have added the ponceau stain as requested. The elevated PARP1 is not due to loading differences however, because both the level of histones and the ponceau staining is similar across the samples. Given the role of PCNA complexes in coordinating lagging strand synthesis it is possible that the accumulation of PCNA is another consequence of the loss of FEN1-dependent OF maturation (Kubota et al Cell Reports, 2015).

3) The authors should utilize RPA staining to substantiate analysis of ssDNA gap formation being specific, this could be combined with EdU analysis to define newly synthesized DNA. On a similar note, blotting for RPA S4/8 phosphorylation should define whether general replication is perturbed with the formation of ssDNA.

This is an interesting experiment and we have conducted these experiments as requested. We find that RPA foci formation is indeed elevated FEN1-/- U2OS cells in S phase, as is RPA phosphorylation, and is exacerbated by PARPi. These data are described on page 7 and are added as Extended Data Fig. 6.

4) The recent paper from the Bartek group (ref#31) indicated that PARP inhibition affects fork speed quite noticeably. That was a major paper in the field and highly relevant to the current manuscript because, if correct, it would alter the interpretation of the current data. The Bartek work is 'dismissed' in a single sentence, which is a bit unsatisfactory. The authors propose that the time period of PARP inhibition might explain the difference. I suggest that they test this proposal directly and, if correct, then their claims would be far stronger.

We have now measured as requested fork rates in DT40 cells over the time course employed by Maya-Mendoza et al. However, surprisingly, we did not see any impact of PARPi under any of the conditions tested. To confirm we conducted these experiments correctly we also employed U2OS cells (the cell line employed by Maya Mendoza et al). Reassuringly, although once again we did not see any impact on fork progression rates of short incubation with PARP inhibitor, we did detect such an impact after the 24-hr time period reported by Maya-Mendoza et al. The impact of PARPi on fork rates thus seems to be both time and cell type dependent. We have modified the text accordingly on Page 8 (highlighted in yellow) and included the new data in Extended Data Fig.3D.

5) In an ideal world, although I realize that these things are laborious and complex to conduct, the authors would strain their fibers for ssDNA and/or analyze nascent ssDNA gap formation using electron microscopy.

These are good ideas, and indeed we have been setting up the EM technology for such experiments. These data confirm the increased presence of single-strand gaps in DT40 cells lacking FEN1 and/or treated with PARPi very closely behind forks, and are added in Fig.4E.

6) In Figure 5B, there should be a statistical comparison between the WT and FEN1-/- samples at each time point. Are they really different?

The problem with adding too many *post hoc* comparisons in ANOVA is that each extra comparison reduces the power/significance of the others (multiple t-tests are not appropriate). However, we have added the requested comparisons and the difference between WT and FEN1-/- cells is significant at the 10 min chase time point.

Decision Letter, first revision:

30th Nov 2021

Dear Keith,

Thank you for submitting your revised manuscript "PARP Inhibition Impedes the Maturation of Nascent DNA Strands During DNA Replication" (NSMB-A45127A). It has now been seen by the original referees and their comments are below. I hope that you will be pleased to see that the reviewers find that the paper has improved in revision, and therefore we'll be happy in principle to publish it in Nature Structural & Molecular Biology, pending minor revisions to comply with our editorial and formatting guidelines.

We are now performing detailed checks on your paper and will send you a checklist detailing our editorial and formatting requirements in about a week. Please do not upload the final materials and make any revisions until you receive this additional information from us.

Please do not hesitate to contact me if you have any questions.

With kind regards,

Beth

Beth Moorefield, Ph.D.
Senior Editor
Nature Structural & Molecular Biology

Reviewer #1 (Remarks to the Author):

The authors have nicely addressed all suggestions from the reviewers and I strongly recommend publication of this excellent manuscript

Reviewer #2 (Remarks to the Author):

The authors have made a considerable effort to address all the comments made by referees. As a result, I feel that the revised manuscript is improved considerably. I would now recommend publication in NSMB.

Final Decision Letter:

14th Feb 2022

Dear Keith,

We are now happy to accept your revised paper "PARP Inhibition Impedes the Maturation of Nascent DNA Strands During DNA Replication" for publication as a Article in Nature Structural & Molecular Biology.

Acceptance is conditional on the manuscript's not being published elsewhere and on there being no announcement of this work to the newspapers, magazines, radio or television until the publication date in Nature Structural & Molecular Biology.

Over the next few weeks, your paper will be copyedited to ensure that it conforms to Nature Structural & Molecular Biology style. Once your paper is typeset, you will receive an email with a link to choose the appropriate publishing options for your paper and our Author Services team will be in touch regarding any additional information that may be required.

After the grant of rights is completed, you will receive a link to your electronic proof via email with a request to make any corrections within 48 hours. If, when you receive your proof, you cannot meet this deadline, please inform us at rjsproduction@springernature.com immediately.

You will not receive your proofs until the publishing agreement has been received through our system.

Due to the importance of these deadlines, we ask that you please let us know now whether you will be difficult to contact over the next month. If this is the case, we ask you provide us with the contact information (email, phone and fax) of someone who will be able to check the proofs on your behalf, and who will be available to address any last-minute problems.

To assist our authors in disseminating their research to the broader community, our SharedIt initiative provides all co-authors with the ability to generate a unique shareable link that will allow anyone (with or without a subscription) to read the published article. Recipients of the link with a subscription will also be able to download and print the PDF.

As soon as your article is published, you can generate your shareable link by entering the DOI of your article here: <a href="http://authors.springernature.com/share">http://authors.springernature.com/share<a>. Corresponding authors will also receive an automated email with the shareable link

Note the policy of the journal on data deposition: http://www.nature.com/authors/policies/availability.html.

Your paper will be published online soon after we receive proof corrections and will appear in print in the next available issue. You can find out your date of online publication by contacting the production team shortly after sending your proof corrections. Content is published online weekly on Mondays and Thursdays, and the embargo is set at 16:00 London time (GMT)/11:00 am US Eastern time (EST) on the day of publication. Now is the time to inform your Public Relations or Press Office about your paper, as they might be interested in promoting its publication. This will allow them time to prepare an accurate

and satisfactory press release. Include your manuscript tracking number (NSMB-A45127B) and our journal name, which they will need when they contact our press office.

About one week before your paper is published online, we shall be distributing a press release to news organizations worldwide, which may very well include details of your work. We are happy for your institution or funding agency to prepare its own press release, but it must mention the embargo date and Nature Structural & Molecular Biology. If you or your Press Office have any enquiries in the meantime, please contact press@nature.com.

You can now use a single sign-on for all your accounts, view the status of all your manuscript submissions and reviews, access usage statistics for your published articles and download a record of your refereeing activity for the Nature journals.

If you have not already done so, we strongly recommend that you upload the step-by-step protocols used in this manuscript to the Protocol Exchange. Protocol Exchange is an open online resource that allows researchers to share their detailed experimental know-how. All uploaded protocols are made freely available, assigned DOIs for ease of citation and fully searchable through nature.com. Protocols can be linked to any publications in which they are used and will be linked to from your article. You can also establish a dedicated page to collect all your lab Protocols. By uploading your Protocols to Protocol Exchange, you are enabling researchers to more readily reproduce or adapt the methodology you use, as well as increasing the visibility of your protocols and papers. Upload your Protocols at www.nature.com/protocolexchange/. Further information can be found at www.nature.com/protocolexchange/about.

An online order form for reprints of your paper is available at https://www.nature.com/reprints/author-reprints.html. Please let your coauthors and your institutions' public affairs office know that they are also welcome to order reprints by this method.

Please note that <i>Nature Structural & Molecular Biology</i> is a Transformative Journal (TJ). Authors may publish their research with us through the traditional subscription access route or make their paper immediately open access through payment of an article-processing charge (APC). Authors will not be required to make a final decision about access to their article until it has been accepted. Find out more about Transformative Journals

<B>Authors may need to take specific actions to achieve

compliance with funder and institutional open access mandates. For submissions from January 2021, if your research is supported by a funder that requires immediate open access (e.g. according to Plan S principles) then you should select the gold OA route, and we will direct you to the compliant route where possible. For authors selecting the subscription publication route our standard licensing terms will need to be accepted, including our self-archiving policies. Those standard licensing terms will supersede any other terms that the author or any third party may assert apply to any version of the manuscript.

In approximately 10 business days you will receive an email with a link to choose the appropriate publishing options for your paper and our Author Services team will be in touch regarding any additional information that may be required.

You will not receive your proofs until the publishing agreement has been received through our system.

If you have any questions about our publishing options, costs, Open Access requirements, or our legal forms, please contact ASJournals@springernature.com

With kind regards,

Beth

Beth Moorefield, Ph.D.
Senior Editor
Nature Structural & Molecular Biology
